# Structural and epitope characterization of anti-DEFA5 monoclonal antibodies clones 1A8 and 4F5 for inflammatory bowel disease subtype diagnostics

**DOI:** 10.1016/j.ijbiomac.2025.148024

**Published:** 2025-10-09

**Authors:** Rabi Thangaiyan, Anil Shanker, Billy R. Ballard, Amosy E. M’Koma

**Affiliations:** a Department of Biochemistry, Cancer Biology, Neuroscience and Pharmacology, Meharry Medical College, Nashville, TN, 37208, USA; b Department of Pathology, Anatomy, and Cell Biology, Meharry Medical College, Nashville, TN37208, USA

**Keywords:** DEFA5, IBD, Epitope mapping, Monoclonal antibodies, Clone 1A8, Clone 4F5

## Abstract

Understanding antigen-antibody interactions is key to advancing immunodiagnostics and therapeutic development. Here, we characterize two mouse monoclonal antibodies, 1A8 and 4F5, specific to human DEFA5, an innate immune effector implicated in differentiating colonic inflammatory bowel disease (IBD) subtypes, particularly Ulcerative colitis, Crohn’s colitis and Indeterminate colitis. Although both antibodies target similar regions of DEFA5, they exhibit distinct binding affinities. Hydrogen/deuterium exchange mass spectrometry (HDX-MS) revealed diffuse protection patterns with partial protection upon antibody binding, suggesting conformational stabilization in DEFA5 rather than discrete epitope recognition. Complementary peptide-based epitope mapping confirmed localized linear or semi conformational binding epitopes. Surface Plasmon Resonance (SPR) confirmed 1:1 binding stoichiometry and high-affinity binding of both antibodies for the DEFA5 epitope RATCYCRTGRCAT. Hybridoma sequencing revealed that both antibodies engage DEFA5 by an extended binding interface involving multiple complementarity-determining regions (CDRs) across both heavy and light chains, indicating structurally distributed mode of antigen recognition. Despite identical hybridoma sequencing, HADDOCK models reveal subtle binding pose differences with DEFA5 engages the VH domain in 1A8, while it interacts with the VL domain in 4F5, suggesting phenotypic variations in domain contributions. This integrative approach provides mechanistic insights into their diagnostic potential for distinguishing IBD subtypes, supporting refined tissue-based and serological assays.

## 1. Introduction

Accurate classification of colonic Inflammatory Bowel Disease (IBD) subtypes is crucial for guiding treatment strategies, but current diagnostic methods often lack specificity, particularly in distinguishing Ulcerative Colitis (UC) from Crohn’s Colitis (CC) and identifying inexact diagnosis of Indeterminate Colitis (IC) cases when the diagnostic classification features for UC and CC are inconclusive. The distinction between UC and CC in otherwise IC into authentic UC and CC is of utmost importance when determining a patient’s candidacy for pouch surgery, the restorative proctocolectomy with ileal pouch-anal anastomosis (RPC-IPAA), the standard curative surgical procedure in the treatment for UC. Further, incorrect diagnosis and treatment carry potential morbidity from inappropriate and unnecessary surgery and cost. The success outcomes of RPC-IPAA and convalescence depend on correct diagnosis [1–3]. Among potential biomarkers, human alpha defensin 5 (DEFA5 alias HD5) has emerged as a promising candidate due to its differential expression patterns in UC and CC, making it a valuable target for diagnostic development [4]. We previously reported that DEFA5 is ectopically expressed in colonic tissues from patients with Crohn’s colitis and some indeterminate colitis and demonstrated that this expression pattern holds high diagnostic value with a positive predictive value of 96 **%** in distinguishing these conditions from ulcerative colitis [5]. DEFA5 expression is markedly elevated in CC, whereas its levels decreased in UC, distinguishing CC from UC with high specificity [4]. These differential expression patterns underscore the potential of DEFA5 as a biomarker for IBD classification, necessitating highly specific antibodies for its detection. Commercially available antibodies fail to consistently differentiate UC from CC, often due to low epitope specificity [4,6–8]. To address this challenge, we recently generated two mouse monoclonal antibodies 1A8 and 4F5 targeting DEFA5 with the goal of delivering enhanced diagnostic and research utility [4]. Western blot confirmed the antibodies’ ability to detect DEFA5 with high specificity and sensitivity. Immunohistochemistry revealed a well-defined DEFA5 expression pattern, validating its tissue distribution. Immunoprecipitation/Western blot further confirmed the antibodies’ strong binding affinity to DEFA5, while ELISA demonstrated the quantitative detection capability of these antibodies, providing a higher sensitive platform than commercial alternatives for measuring DEFA5 levels in UC, CC, and IC patient biopsy samples.

Human α defensin (DEFA5) is an essential antimicrobial peptide primarily secreted by Paneth cells in the small intestine, where it plays a crucial role in maintaining gut immunity and epithelial barrier integrity. DEFA5 is initially synthesized as an inactive precursor, proHD5, and stored in Paneth cell granules. Through cholinergic activation by carbamylcholine, proHD5 is secreted into the intestinal lumen, where it undergoes proteolytic cleavage by trypsin at R62↓A63, yielding the predominant active form of HD5 [9]. Alternative processing sites have also been identified, leading to additional HD5 variants such as HD5 (37–94), indicating multiple regulatory mechanisms in defensing activation [10]. Functionally, DEFA5 exhibits broad spectrum antimicrobial, antiviral, and antifungal activity, disrupting microbial membranes [11–13], and selectively shaping the gut microbiota by inhibiting pathogenic bacteria while sparing commensals [11,14]. Beyond its antimicrobial properties, DEFA5 regulates epithelial barrier integrity, and immune responses, influencing gut homeostasis [11,15,16]. Dysregulation of DEFA5 has been linked to dysbiosis, a microbial imbalance that contributes to IBD, metabolic disorders, and colorectal cancer [17]. Moreover, recent studies suggest that DEFA5 also interacts with host metabolites, further influencing intestinal immune homeostasis [18].

In this study, we employed a combination of hydrogen/deuterium exchange mass spectrometry (HDX-MS), peptide mapping, surface plasmon resonance (SPR), and hybridoma sequencing to identify and validate the epitope region recognized by clones 1A8 and 4F5. We further compared their structural features and binding affinities to explain their differential behavior in DEFA5-based diagnostic assays. These findings contribute to the development of epitope specific diagnostic reagents for both serological and tissue-based discrimination between UC, CC, and IC into UC and CC, addressing a critical unmet need in IBD diagnostics.

## 2. Materials and methods

### 2.1. Expression and purification of DEFA5 protein

**Fig. 1.**
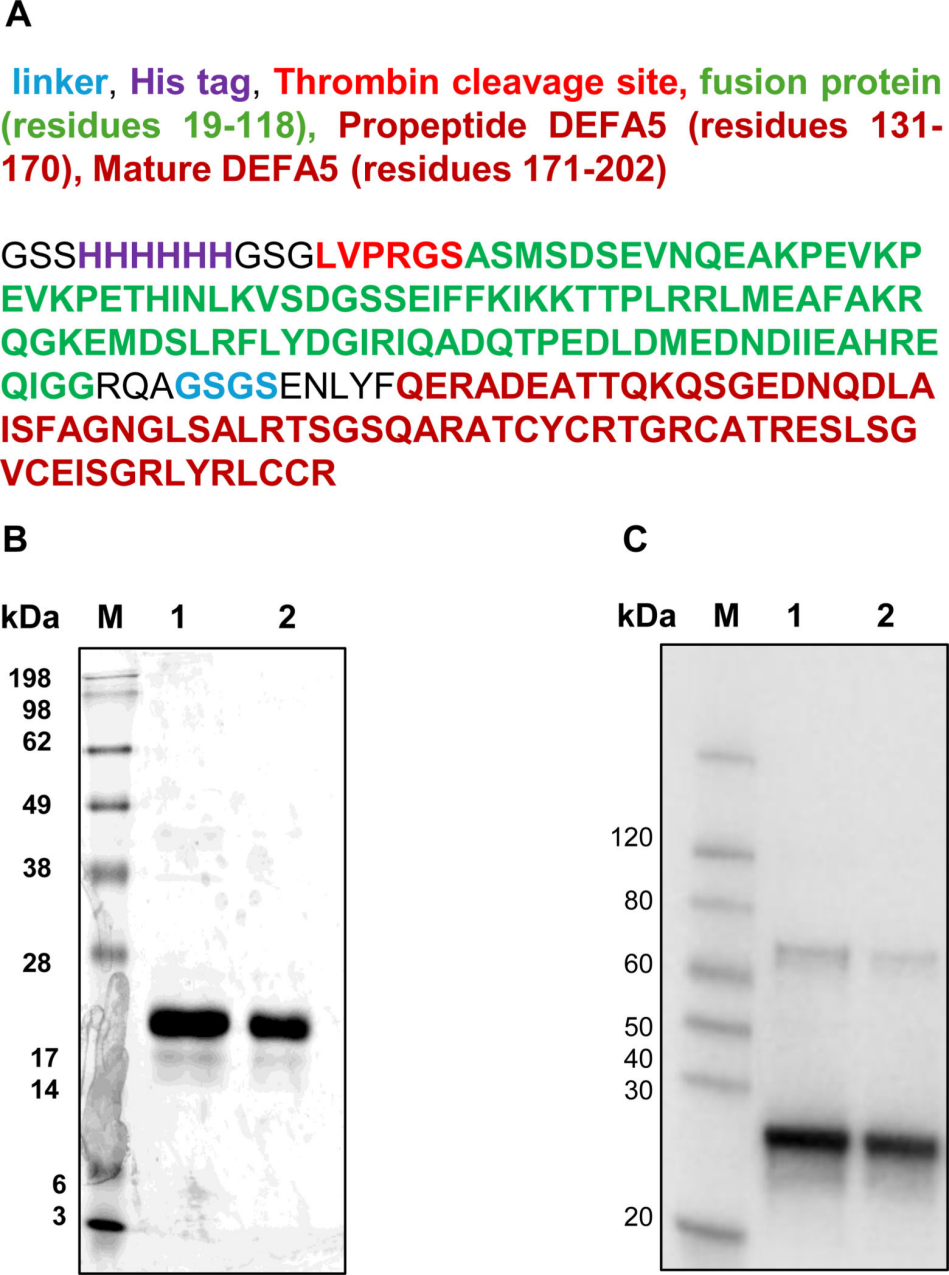
SDS-PAGE and Western blot analysis of recombinant DEFA5-His protein. Schematic of recombinant DEFA5 construct with N-terminal His₆-tag, thrombin cleavage site, fusion partner, linker, and DEFA5, showing colour-coded domains with amino acid lengths (B) SDS-PAGE of DEFA5-His protein from *E. coli*, assessing purity and expression. (C) Western blot with anti-His antibody confirming His-tagged DEFA5. Lane M: molecular weight markers; Lane 1: 1 μg; Lane 2: 0.5 μg.

The codon-optimized human α-defensin 5 (DEFA5) gene, encoding residues 23–94 of UniProt Q01523 (encompassing the propeptide and mature peptide), was custom-synthesized and cloned into the pET-30a expression vector. The construct was designed in-frame with an N-terminal His6 tag, a thrombin cleavage site, a soluble fusion partner, a TEV protease cleavage site, and a synthetic linker, with the DEFA5 sequence positioned at the C-terminus. The complete construct comprises 202 amino acids with the following sequence: GSSHHHHHHGSGLVPRGSASMSDSEVNQEAKPEVKPEVKPE-THINLKVSDGSSEIFFKIKKTTPLRRLMEAFAKRQGKEMDSLR-FLYDGIRIQADQTPEDLDMEDNDIIEAHREQIGGRQAGSGSENLYFQER-ADEATTQKQSGEDNQDLAISFAGNGLSALRTSGSQARATCYCRTGRCA-TRESLSGVCEISGRLYRLCCR. The calculated molecular weight of the full construct is 22.35 kDa, with the DEFA5 portion contributing to 7.77 kDa [Fig. 1A]. A verified glycerol stock of *E. coli* BL21 Star^™^ (DE3) containing the DEFA5 construct was used for recombinant protein production. Cultures were grown in LB medium with 50 μg/mL kanamycin at 37 °C and 220 rpm for 6 h, followed by induction with 1 mM IPTG for 4 h at 37 °C and an additional 16 h at 15 °C. Cells were harvested at OD_600_ = 1.2 by centrifugation at 12,000 rpm for 2 min. Pellets were resuspended in lysis buffer, sonicated, and centrifuged to collect the soluble fraction. DEFA5 protein was purified using Ni^2+^ - affinity chromatography followed by Superdex 75 column. The purified protein was sterilized using a 0.22 μm filter and stored in aliquots at −80 °C. Protein expression and purity were assessed by SDS-PAGE. Western blotting was performed using an anti-His antibody to confirm the presence of the His-tagged DEFA5 protein.

### 2.2. HDX-MS analysis

To prepare samples for hydrogen-deuterium exchange mass spectrometry (HDX-MS), four different sample conditions were generated using newly prepared batches of DEFA5 and its potential binding partners. Controls included DEFA5 mixed with PBS (condition 1). 300 μL of DEFA5 (0.6 mg/mL, 60 μM) was mixed with 120 μL of sc-53,997 DEFA5 antibody (2 mg/mL, 13.3 μM) and incubated on ice for 2 h to allow complex formation. Subsequently, samples were prepared by combining 106.6 μL of the DEFA5 + sc-53,997 complex with 26.65 μL of either PBS (condition 2), 1A8 (condition 3), or 4F5 (condition 4), where 1A8 and 4F5 were used at concentrations of 5.1 mg/mL (34 μM) and 7.2 mg/mL (48 μM), respectively. For each HDX-MS reaction, 10 μL of the prepared sample was mixed with 40 μL of deuterated buffer (1× PBS containing 500 mM NaCl, pH 7.0 in D₂O), initiating the deuterium exchange. The reaction was carried out at four time points, namely 10 s, 100 s, 1000 s, and 1 h. At each time point, the exchange was quenched by adding 60 μL of quench buffer (8 M urea, 500 mM TCEP, pH 2.5), denaturing the proteins and stopping further exchange. Immediately following quenching, 140 μL of glycine buffer (100 mM, pH 2.5) was added to stabilize the sample for injection into the ion mobility mass spectrometer. All samples were run in triplicate to ensure reproducibility and statistical significance. This experimental setup was designed such that DEFA5 was first incubated with commercially available sc-53,977 DEFA5 antibody from Santa Cruz Biotechnology to induce potential conformational changes that could potentially expose cryptic epitope regions.

### 2.3. Peptide design and synthesis

To ensure comprehensive mapping and to detect any potential cross-reactivity of 1A8 and 4F5 with non-DEFA5 sequences such as His6 tag, thrombin cleavage site, soluble fusion partner, TEV protease cleavage site, or synthetic linker, we designed an overlapping peptide library covering the entire 202 amino acids. A library of 64 custom-synthesized overlapping peptides was obtained from GenScript (Piscataway, NJ, USA) [Table 1]. Each peptide was 15 aminoacids in length with a 12-aminoacid overlap and a 3-aminoacid offset to ensure complete coverage of the full-length DEFA5 protein sequence. All peptides were verified to have a purity greater than 95 % as determined by HPLC. These peptides were used in indirect ELISA assays to identify antibody-binding epitopes.

### 2.4. Peptide ELISA mapping

Peptide ELISA was performed to screen for specific antigenic regions of DEFA5. Each peptide was diluted to a final concentration of 5 μg/mL in ELISA coating buffer, and 100 μL of the diluted peptide solution was added in triplicate wells of a 96-well high binding ELISA plate (R&D SYSTEMS). Plates were incubated at 4 °C overnight to allow for peptide adsorption. Following incubation, plates were washed three times with PBST and blocked with 5 % BSA in PBS at 37 °C for 2 h to minimize non-specific binding. After blocking, wells were incubated with either monoclonal antibody clone 1A8 or 4F5 diluted in PBST containing 1 % BSA. Plates were incubated at 37 °C for 2 h. After washing, wells were treated with 1:5000 diluted horseradish peroxidase (HRP)-conjugated goat ant-mouse IgG secondary antibody for 1 h at 37 °C. After final washes, 50 μL of TMB substrate (3,3′,5,5′-tetramethylbenzidine) was added per well and the reaction was settled for 10–15 min in the dark at room temperature. The enzymatic reaction was stopped by adding 50 μL of stop solution to each well. The absorbance was measured at 450 nm using a microplate reader. OD values were analyzed to determine peptide reactivity.

### 2.5. Surface plasmon resonance analysis

SPR experiments were conducted on a Biacore 8 K instrument (Cytiva) using a Series S Sensor Chip SA for high-affinity peptide-antibody interaction studies. The biotinylated DEFA5 epitope peptide (Biotin-RATCYCRTGRCAT, 1 μg/mL) was immobilized onto flow cell 2 at low surface density (~95 RU) to minimize mass transport effects and preserve binding kinetics. Flow cell 1 remained unmodified and was used as a reference. Prior to immobilization, the chip surface was conditioned with three 1-min injections of 1 M NaCl in 50 mM NaOH, followed by a wash with 50 % isopropanol in 1 M NaCl and 50 mM NaOH. The assay was performed at 25 °C using HBS-EP^+^ running buffer at a constant flow rate of 30 μL/min. Monoclonal antibodies 1A8 and 4F5 were diluted in running buffer and injected in a two-fold serial dilution series (125, 62.5, 31.25, 15.63, and 7.81 nM). Each injection consisted of a 120-s association phase followed by a 180-s dissociation phase. These conditions were selected based on preliminary evaluations of binding stability and dissociation characteristics of the antibody-peptide interactions. Between cycles, the chip surface was regenerated using 10 mM glycine-HCl (pH 1.5) after confirming its compatibility with the peptide ligand in preliminary regeneration scouting tests to avoid potential ligand denaturation or loss of binding activity. SPR data were processed using Biacore 8 K Evaluation Software version 5.0. A double-referencing approach was applied by subtracting the response from flow cell 1 and buffer-only blank cycles. Binding kinetics were analyzed using a global fit to a 1:1 L binding model to derive the association rate constant (ka), dissociation rate constant (kd), and equilibrium dissociation constant (KD).

### 2.6. Fine epitope mapping by mutagenesis and N-terminal deletion

To refine the epitope structure recognized by 1A8 and 4F5 monoclonal antibodies, synthetic peptide variants based on the identified reactive motif ARATCYCRTGRCATR were generated. Each residue within the sequence was systematically substituted with alanine to assess its role in antibody binding. In addition, alanine residues were replaced with glycine to evaluate the contribution of side-chain flexibility. A separate peptide variant was synthesized with a single deletion of the N-terminal alanine to determine its necessity for antibody recognition. All peptides were synthesized by GenScript at >95 % purity and the binding reactivity of each peptide mutant to 1A8 and 4F5 antibodies was tested using ELISA, as described in the previous section. The data generated from these assays provided valuable insights into which specific residues are essential for 1A8 and 4F5 recognition and helped define the precise epitope regions on the DEFA5 protein targeted by both antibodies.

### 2.7. ELISA based estimation of binding affinity

To evaluate the binding affinities of the monoclonal antibodies 1A8 and 4F5 for DEFA5, an indirect ELISA was performed to estimate the dissociation constant (KD). ELISA plates were coated with recombinant DEFA5 protein at a concentration of 100 ng/mL in carbonate-bicarbonate buffer (pH 9.6) and incubated overnight at 4 °C. After blocking with 5 % BSA in PBS-T (PBS with 0.05 % Tween-20), wells were incubated with serial two-fold dilutions of 1A8 and 4F5 antibodies ranging from 250 nM to 3.9 nM. Following incubation at room temperature for 1 h, plates were washed and probed with horseradish peroxidase (HRP)-conjugated anti-mouse IgG secondary antibody. Colorimetric detection was performed using TMB substrate, and absorbance was measured at 450 nm. Nonlinear regression analysis of the binding curves was performed using a one-site specific binding model in GraphPad Prism to calculate the KD values for each antibody.

### 2.8. Hybridoma sequencing

Hybridoma sequencing was conducted to clone, sequence, and analyze the antibody variable and constant regions of the heavy and light chains of the 1A8 and 4F5 monoclonal antibody (mAb) hybridoma clones to fully characterize the antibody sequences by combining 5’ RACE (Rapid Amplification of cDNA Ends) PCR and 3’ RACE PCR at Syd Labs, Inc. (Hopkinton, MA, USA). The total RNA was extracted from the hybridoma cells using Zymo RNA microprep kit (Irvine, CA, USA). The mRNA was then reverse transcribed into complementary DNA (cDNA) for amplification. 5’ RACE PCR was employed to amplify the variable regions of the heavy and light chains of the mAbs, which contain the complementarity-determining regions (CDRs). These CDRs are critical for antigen binding and are located within variable regions. The PCR products obtained from 5’ RACE PCR were used to clone and sequence the variable region sequences and accurately identify the CDRs using IMGT/V-QUEST (https://www.imgt.org) and other sequence alignment tools. To ensure we captured the full-length antibody genes, including the constant regions, we also performed 3’ RACE PCR. This method targeted the 3′ end of the mRNA, amplifying the constant regions of both the heavy and light chains. The PCR products from both 5′ and 3’ RACE reactions were respectively cloned into the pSL003 plasmid vector (Syd Labs). Plasmid DNA was isolated from bacterial colonies, and Sanger sequencing of 20 clones per hybridoma sample was performed by Quintara Biosciences, Inc. (Boston, MA, USA). The sequencing data were then analyzed to identify the CDRs within the variable regions and to assemble the complete sequence of the antibodies, including both the variable and constant regions. To validate the sequencing data, the variable heavy (VH) and variable light (VL) chain constructs of 1A8 and 4F5 were cloned and expressed in a controlled HEK293 cell expression system. The recombinant antibodies were purified from the cell culture supernatant using Protein A affinity chromatography. The purified antibodies were tested for binding affinity to recombinant DEFA5 using indirect ELISA and to DEFA5 in IBD biopsy samples using Sandwich ELISA.

### 2.9. Structural context of the DEFA5 epitope and antibody modelling

To provide structural context, the epitope (RATCYCRTGRCAT) was first mapped onto the NMR structure of DEFA5 (PDB ID: 1ZMP) using PyMOL, allowing visualization of the epitope within the experimentally resolved portion of the protein. To capture the full sequence context, including regions predicted to be intrinsically disordered, the AlphaFold-predicted structure of DEFA5 (AF-Q01523) was analyzed. This model was annotated in PyMOL to highlight the epitope (residues 62–74) and to identify areas of structural disorder. To explore the molecular basis of antibody binding, antibody–antigen complexes were modeled using AlphaFold-Multimer (ColabFold implementation) with paired input sequences for the variable heavy (VH) and light (VL) chain sequences of 1A8 and 4F5 Fabs and individually fused to a full-length model of mature DEFA5. The N-terminal residue R1, normally part of the propeptide and absent from the mature DEFA5 was added, since the epitope spans residues RATCYCRTGRCAT (62–74), and generated twenty complex models per antibody. Two high-ranking models for 1A8-DEFA5 and 4F5-DEFA5 were selected based on optimized buried surface area, hydrogen bonds, salt bridges, and antibody-antigen interface quality, as determined by PyMOL analysis, which approximate binding affinity of 1A8 (43.83 nM) and 4F5 (54.97 nM), were subsequently refined using HADDOCK2.4 with key mutagenesis-identified residues (R10, C6, C11) defined as active restraints. The final top-ranked HADDOCK models were analyzed using PDBePISA to characterize the binding interfaces and identify specific interactions.

### 2.10. Intact ESI-MS and glycoform analysis

Purified monoclonal antibodies 1A8 and 4F5 were buffer exchanged into 100 mM ammonium acetate (pH 6.8) using Zeba Spin Desalting Columns (Thermo Fisher Scientific) to remove non-volatile salts. For complex formation, DEFA5 protein was mixed with either 1A8 or 4F5 at a 1:1 M ratio and incubated on ice for 10 min prior to analysis. Intact mass analysis was performed using a Waters Xevo G2-XS QTof mass spectrometer equipped with an electrospray ionization source. The samples were directly infused at a flow rate of 5 μL per minute. The instrument was operated in positive ion mode with a capillary voltage of 3.0 kV, cone voltage of 50 V, source temperature of 120 °C, and desolvation temperature of 275 °C. Mass spectra were acquired over an *m*/*z* range from 500 to 8000. Raw spectra were processed and deconvoluted using the MaxEnt1 algorithm available in the MassLynx version 4.2 software. For glycoform analysis, the deconvolution was performed over a mass range of 140,000 to 160,000 Da with a resolution of 1.0 Da/channel. Glycoform and post-translational modification assignments were made based on characteristic mass shifts, −146 Da (loss of fucose, G0), −162 Da (loss of galactose, G0F), +162 Da (addition of galactose, G2F), +128 Da (C-terminal lysine), and − 16 Da (oxidation). The relative abundance of each species was calculated from the normalized intensity of its deconvoluted peak.

## 3. Results

### 3.1. Preparation of recombinant DEFA5 protein

Recombinant DEFA5 protein expression in *E. coli* was induced with IPTG and analyzed in both the cell supernatant and pellet fractions following ultrasonication. When cells were cultured in LB medium supplemented with L-arabinose and induced with IPTG, recombinant DEFA5 expression was successfully enhanced. Furthermore, expression at a reduced temperature (15 °C) led to increased solubility, with a greater proportion of DEFA5 protein detected in the supernatant under these conditions. Purification of the His-tagged DEFA5 protein was performed using Ni-NTA affinity chromatography. Non-specifically bound proteins were removed using a buffer containing 20 mM imidazole, and the target protein was eluted with a buffer containing 100 mM imidazole. Ultrafiltration was subsequently employed to concentrate the eluted protein. SDS-PAGE analysis under reducing conditions with β-mercaptoethanol and Coomassie blue staining revealed a prominent band migrating near 22 kDa (Fig. 1B). Western blotting using an anti-His tag antibody confirmed the presence of His-tagged recombinant protein closely migrating prominent band in the same region, comparable to the Coomassie-stained band (Fig. 1C), confirmed this band as the full-length construct (calculated MW 22.35 kDa, including the 7.77 kDa DEFA5 portion). These findings contrast with our previous non-reducing SDS-PAGE results, the ~13 kDa species in non-reducing conditions [4] is the compact, disulfide-bonded form of the full His-tagged DEFA5 construct (22.35 kDa), migrating faster, with a ~ 26 kDa band (likely dimer), due to reduced SDS binding, a common trait of defensins.

### 3.2. HDX-MS indicates conformational shielding of DEFA5 epitope by 1A8 and 4F5

Peptide coverage map of DEFA5 generated by HDX-MS reveals high sequence coverage across the protein backbone, ensuring reliable detection of structural dynamics upon antibody binding (Fig. S1). Deuterium uptake plots comparing DEFA5 alone and DEFA5 in complex with 1A8 or 4F5 antibodies revealed localized regions of reduced deuterium incorporation, indicative of diffuse and partial protection patterns. These reductions were observed primarily in two overlapping peptides, residues 54–66 (LRTSGSQARATCY) for 1A8 and residues 52–66 (SALRTSGSQARATCY) for 4F5 (Fig. S2). Notably, DEFA5 alone did not form detectable complexes with either 1A8 or 4F5 antibodies in solutions in the HDX-MS experiments (data not shown). Therefore, in this HDX-MS study, 1A8 and 4F5 were applied following pre-binding of DEFA5 by the sc-53,997 monoclonal antibody (Santa Cruz Biotechnology). HDX-MS shows sc-53,997 antibody binds DEFA5 at peptide 67–83, CR**TGRCATRE**SLSGVCE (~48–61 % D vs. 68–125 % D in apo, EX1-to-EX2 shift), confirming the epitope (core TGRCATRE**)**, and induces allosteric stabilization at 43–51, AISFANGL (~63–72 % D, EX1-to-EX2 kinetic transition) (Fig. S3), suggesting structural rearrangements that likely enable 1A8/4F5 binding, consistent with IP/WB and ELISA, which well documented that sc-53,997 antibody binding promote 1A8/4F5 binding [4]. SPR with sc-5–53,997 (10 μg/mL, ~676.2 RU capture) and DEFA5 (372.02–5952.32 nM, 120 s/180 s) shows Rmax 947.2 RU and ~ 70 RU dissociation (molar ratio < 0.1), supporting 1:2 stoichiometry (Fig. S4A). Affinity data suggest bivalent binding, indicating sc-53,997 binds multiple DEFA5 molecules, potentially optimizing 1A8/4F5 interactions (Fig. S4B). The sc-53,997 antibody likely induced conformational rearrangements or exposed otherwise buried regions of DEFA5, facilitating subsequent interaction with 1A8 and 4F5. While 1A8 and 4F5 exhibit epitope engagement upon secondary binding to the DEFA5–sc-53,997 complex, the resulting protection patterns are diffuse and not sharply localized to a single conformational site. Instead, these findings point to interactions with flexible or extended surface regions of DEFA5 that may contribute to antibody recognition but are not independently sufficient to define a functional, high-affinity binding epitope in the absence of conformational priming. To evaluate whether the regions identified by HDX-MS were sufficient for specific antibody recognition, Surface Plasmon Resonance (SPR) analysis was performed using synthetic peptides spanning residues 54–66 (LRTSGSQARATCY) for 1A8 and 52–66 (SALRTSGSQARATCY) for 4F5. SPR sensograms revealed weak binding affinities, with calculated dissociation constants (KD) of 303 μM for 1A8 and 4800 μM for 4F5, respectively (Fig. S5). These findings confirm that while these regions may contribute to antibody interaction, they are insufficient on their own to support high-affinity binding and therefore do not represent the full functional epitopes required for strong and specific recognition. Although the precise binding interface could not be sharply localized by HDX-MS, the protected region encompassed the RATCY motif, which was subsequently confirmed to be part of the minimal epitope by peptide mapping.

### 3.3. Epitope identification by synthetic peptide mapping

**Fig. 2.**
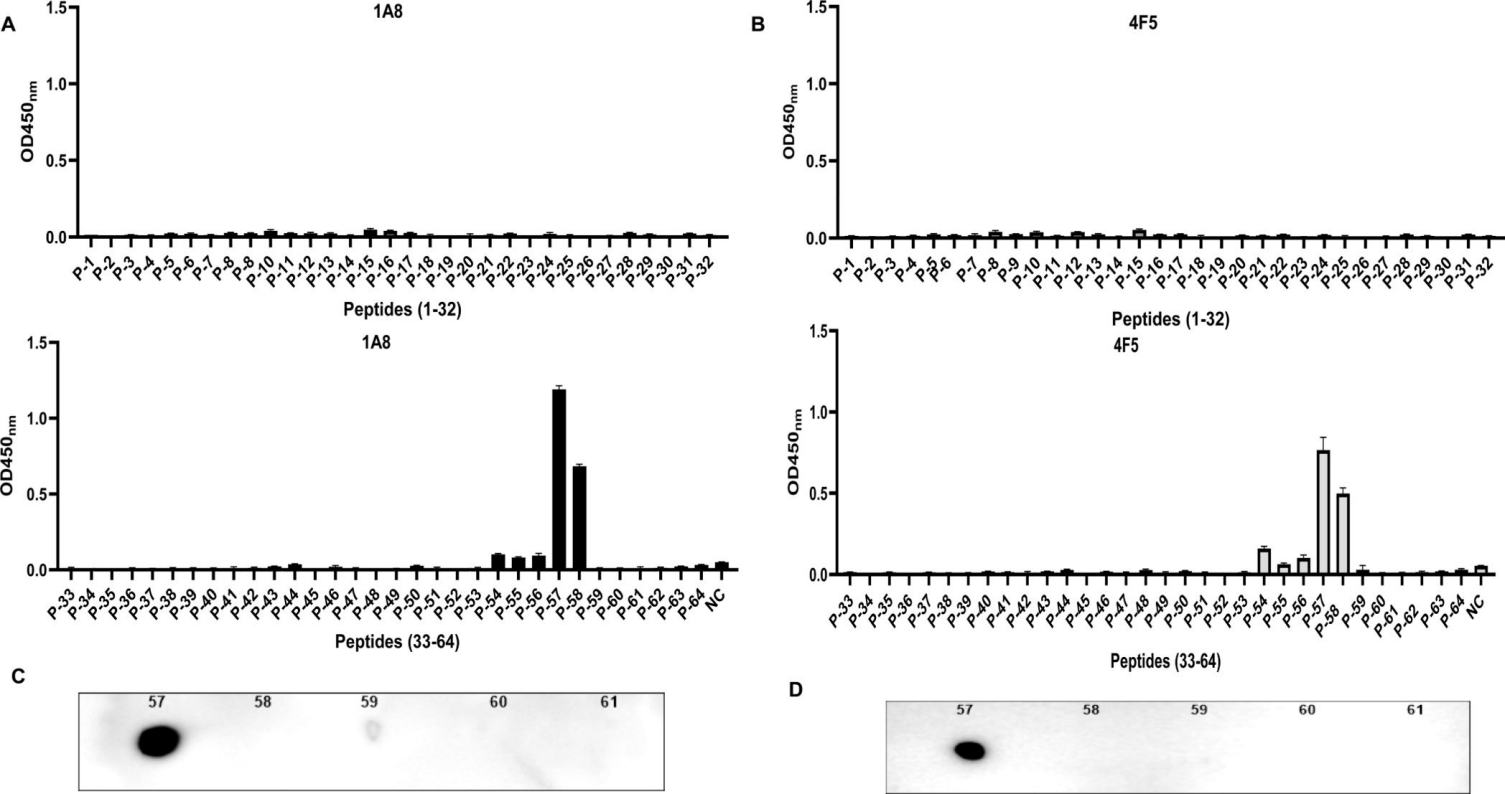
Epitope mapping of mAbs 1A8 and 4F5 using synthetic DEFA5 peptides. (A-B) Indirect ELISA results showing the binding reactivity of mAb 1A8 (A, dark gray) and mAb 4F5 (B, light gray) against a library of 64 synthetic peptides spanning the entire DEFA5 sequence, including the His-tag, linker, TEV site, and fusion protein region. (C–D) Dot blot showing the binding reactivity of antibody 1A8 (C) and 4F5 (D) for peptides 57–61.

Given the HDX-MS and SPR data indicated that the 52–66 and 54–66 peptide segments alone were insufficient for high-affinity antibody binding, we next employed a synthetic peptide mapping approach to define the fine epitope recognized by 1A8 and 4F5. A total of 64 overlapping peptides, each offset by three amino acids, were designed and synthesized based on the full-length amino acid sequence of DEFA5 (Table 1). Each peptide was evaluated for antibody binding using an indirect ELISA. The results demonstrated that both 1A8 and 4F5 monoclonal antibodies specifically recognized peptide No. 57, which corresponds to amino acids 61–75 of DEFA5 with the sequence ARA-TCYCRTGRCATR (Fig. 2A and B). No other peptides in the library showed comparable reactivity, confirming the high selectivity of both clones for this region. To further confirm these findings, a dot-blot assay was performed using the peptide. Consistent with the ELISA results, peptide No. 57 also showed strong reactivity with both 1A8 and 4F5 antibodies in the dot-blot assay, confirming this region as a shared linear B cell epitope recognized by both monoclonal antibodies (Fig. 2C and D). P-58 shows moderate binding in ELISA to both antibodies (Fig. 2A and B), but no detectable signal in dot blot (Fig. 2C and D), suggesting that ELISA is more sensitive to detect weaker peptide-antibody interactions for peptide 58.

### 3.4. Fine epitope delineation by N-terminal deletion and mutagenesis

**Fig. 3.**
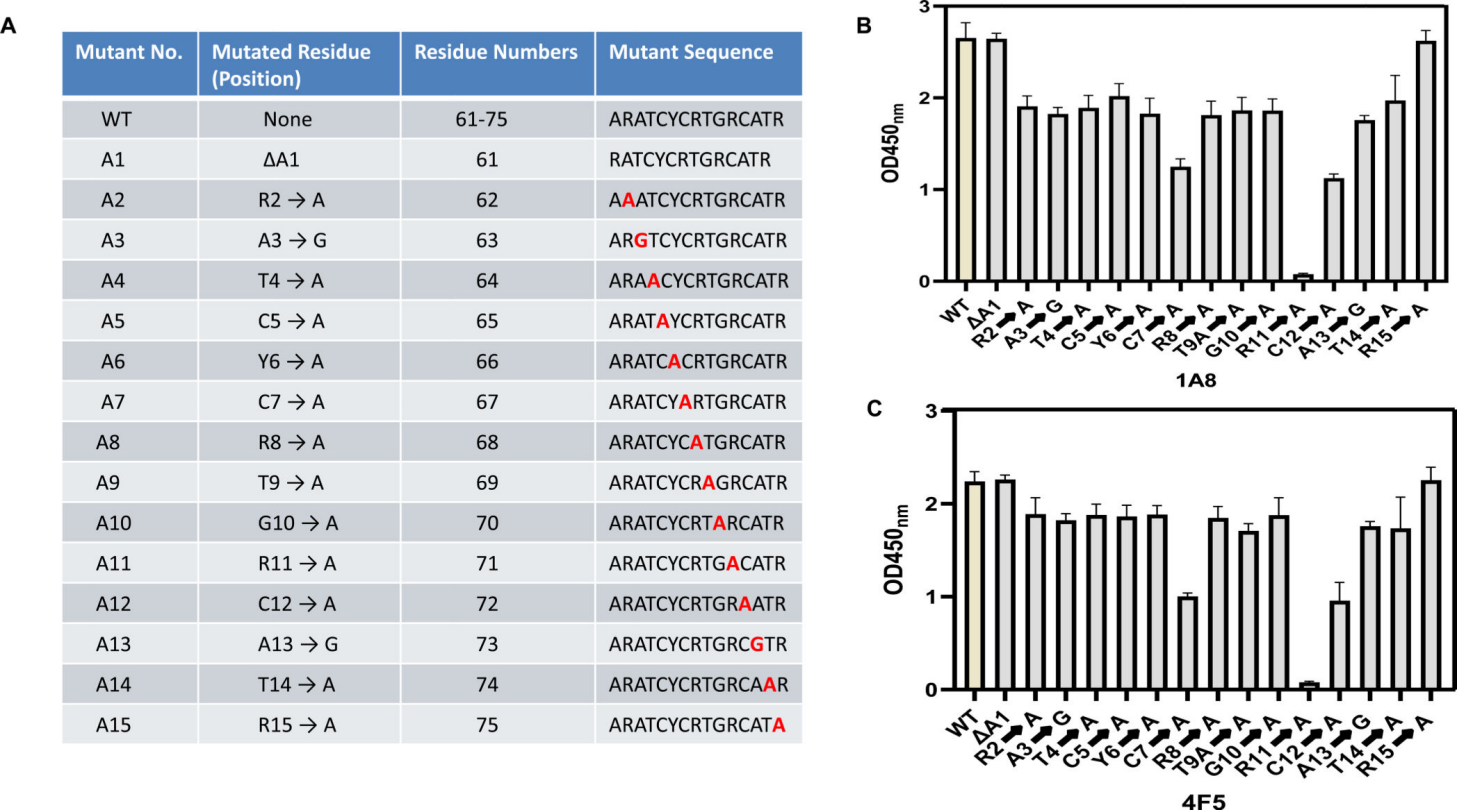
Fine mapping of the DEFA5 peptide epitope. (A) Schematic of the peptide variants showing alanine/glycine substitutions and N-terminal truncation of the peptide sequence ARATCYCRTGRCATR. Residue numbers correspond to the full-length DEFA5 sequence (UniProt Q01523), and the mutated residue in each sequence is highlighted in red within the sequences. (B–C) Indirect ELISA demonstrating the binding of monoclonal antibodies (B) 1A8 and (C) 4F5 to the alanine/glycine substitution and N-terminal deletion mutants.

To determine the critical amino acid residues essential for antibody recognition, single N-terminal deletion, alanine-scanning and glycine substitution was performed on the parent epitope sequence ARA-TCYCRTGRCATR recognized by 1A8 and 4F5. In this approach, each residue within the epitope was individually substituted with alanine or glycine and the resulting mutant peptides were tested for antibody binding using indirect ELISA (Fig. 3A). As illustrated in Fig. 3A, the substitution of specific residues with alanine namely Arg62 (R62), Thr64 (T64), Cys65 (C65), Tyr66 (Y66), Cys67 (C67), Arg68 (R68), Thr69 (T69), Gly70 (G70), Arg71 (R71), Cys72 (C72), and Thr74 (T74) resulted in a significant or complete loss of binding by both mAbs 1A8 and 4F5 (Fig. 3B and C). This loss of reactivity indicates that these residues are essential for antibody recognition and represent the core functional epitope within the region. Additionally, glycine substitutions at two naturally occurring alanine residues Ala63 (A63) and Ala73 (A73) reduced reactivity (Fig. 3B and C), suggesting that even minor alterations to side-chain structure or rigidity can disrupt antibody interaction. Notably, deletion of the N-terminal Ala61 and substitution of Arg75 (R75) with alanine did not impair antibody binding (Fig. 3B and C). Based on these results, the core binding epitope for mAbs 1A8 and 4F5 is defined as RATCYCRTGRCAT (residues 62–74). These results provide a detailed molecular understanding of the antibody-epitope interaction, highlighting distinct but overlapping core binding residues for 1A8 and 4F5 monoclonal antibodies. This information may be pivotal for future structural studies and diagnostic antibody engineering.

### 3.5. SPR confirms RATCYCRTGRCAT as the fine epitope recognized by 1A8 and 4F5

**Fig. 4.**
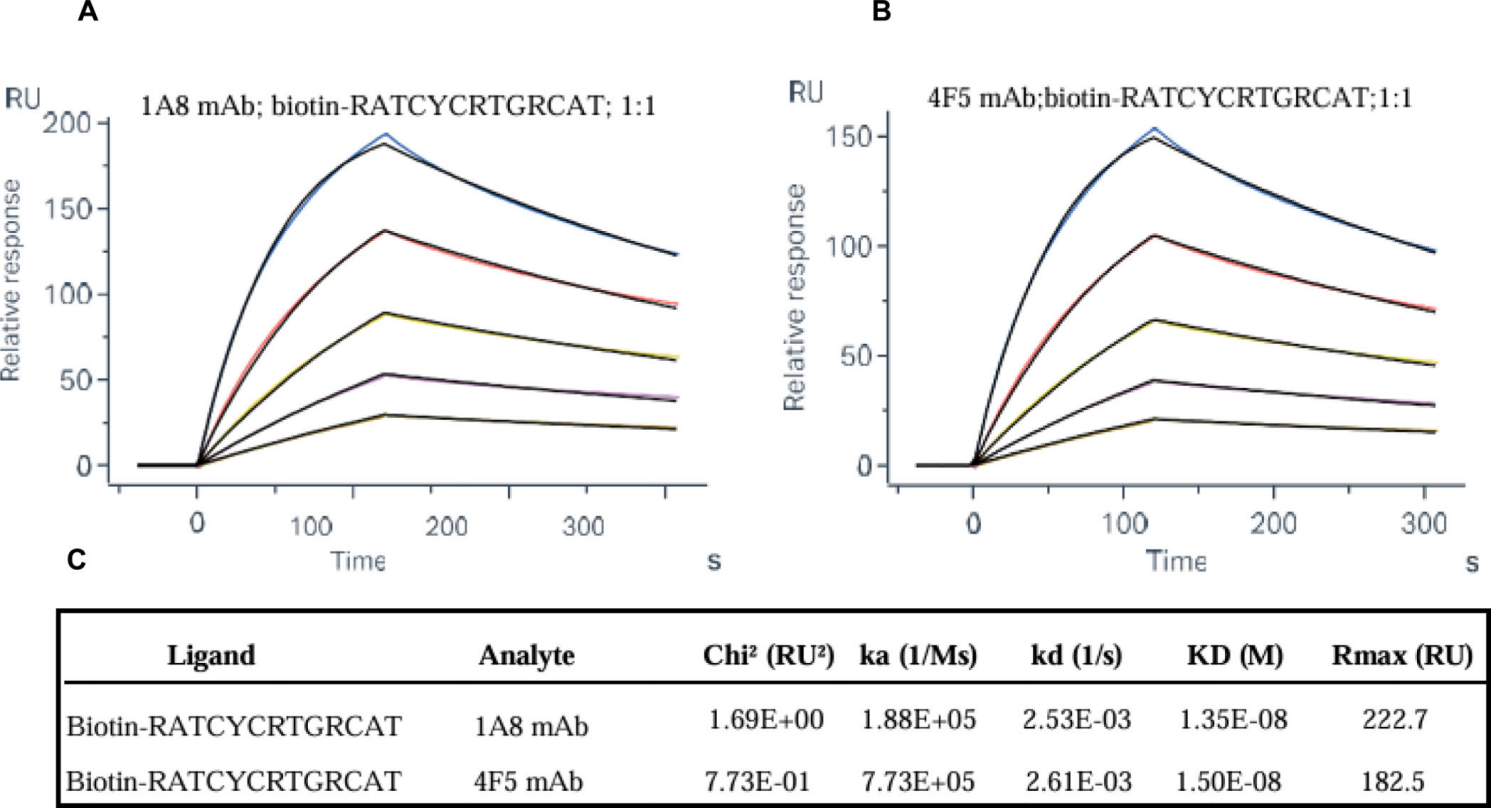
SPR binding analysis of mAbs 1A8 and 4F5 to the RATCYCRTGRCAT peptide. (A-B) Representative sensorgrams for (A) 1A8 and (B) 4F5 show binding responses for serial two-fold dilutions of each antibody (7.81, 15.63, 31.25, 62.5, 125 nM). (C) Summary of the binding kinetics and affinity constants derived from the sensograms.

To precisely define the epitope region recognized by the monoclonal antibodies 1A8 and 4F5, we performed Surface Plasmon Resonance (SPR) analysis using the synthetic peptide RATCYCRTGRCAT, validated as the fine epitope region through mutagenesis and a single N-terminal deletion. The peptide was biotinylated and immobilized on a streptavidin-coated sensor chip to ensure consistent orientation and stable surface presentation. SPR binding assays were conducted by injecting serial dilutions of 1A8 and 4F5 over the immobilized peptide. Both antibodies exhibited strong, concentration-dependent binding, with clear association and dissociation phases. Kinetic analysis using a 1:1 L binding model yielded equilibrium dissociation constants (KD) of 13.5 nM for 1A8 and 15.0 nM for 4F5, confirming high-affinity interactions (Fig. 4A and B). The nanomolar affinities observed by SPR further support the specificity of these interactions, reinforcing the diagnostic utility of this peptide for future ELISA-based assays and epitope-focused applications in inflammatory bowel disease research.

### 3.6. Differential binding affinity of 1A8 and 4F5 to DEFA5

**Fig. 5.**
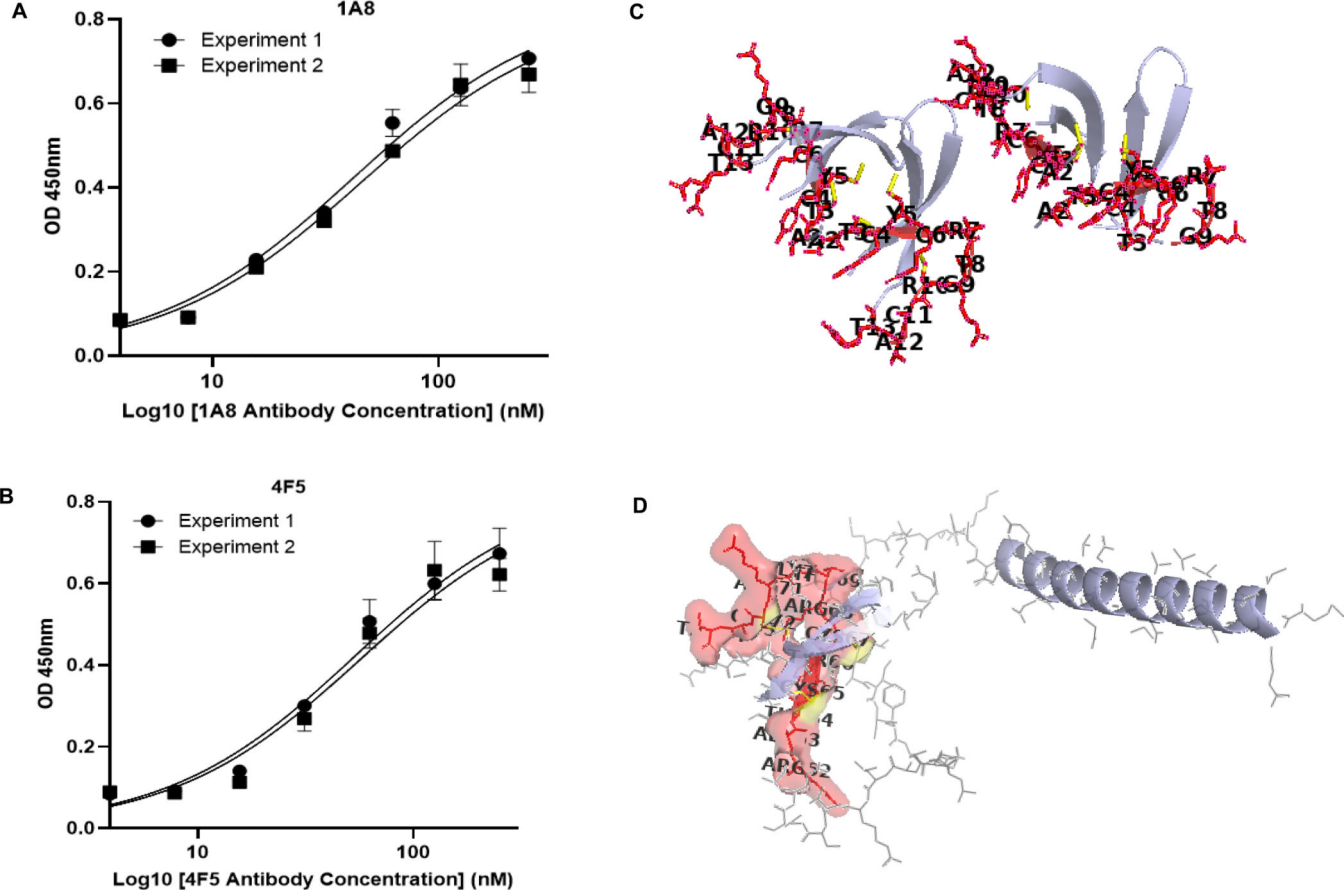
Indirect ELISA for 1A8 and 4F5 affinity. (A-B) Binding curves for (A) 1A8 and (B) 4F5 were generated from two replicates by plotting OD against log antibody concentration (250–3.9 nM). Affinity constants (KD) were determined by nonlinear regression (one-site binding model). Binding affinity between 1A8 and 4F5 is significantly different (*p* = 0.0409, unpaired *t*-test). (C) Epitope (RATCYCRTGRCAT) mapped onto the mature DEFA5 NMR structure (PDB: 1ZMP). Key epitope residues (A2, T3, C4, Y5, R6, G8, R9, C11, A12, and T13) are labeled in black, with the disulfide bond C6-C11 highlighted by a yellow line. Residue R1 is excluded as it is cleaved during maturation, the mature DEFA5 begins at A2 (A63). (D) Epitope mapping of RATCYCRTGRCAT on the AlphaFold2-predicted DEFA5 structure (AF-Q01523-F1), with epitope residues (residues A2, T3, C4, Y5, R6, G8, R9, C11, A12, and T13) shown in red/yellow near β2 strand and magenta (residue R1) in the disordered N-terminal region.

Indirect ELISA was employed to determine the binding affinities (KD values) of the 1A8 and 4F5 monoclonal antibodies to DEFA5. Plates were coated with 100 ng/mL of recombinant DEFA5, and serial two-fold dilutions of each antibody (250 nM to 3.9 nM) were tested. For 1A8, experiment 1 yielded a KD of 42.15 nM (95 % CI: 35.46–50.14 nM), and experiment 2 yielded a KD of 45.52 nM (95 % CI: 37.40–55.46 nM) (Fig. 5A). For 4F5, the corresponding KD values were 53.37 nM (95 % CI: 40.95–69.89 nM) and 56.57 nM (95 % CI: 39.86–80.90 nM), respectively (Fig. 5B). Collectively, these results confirm that both 1A8 and 4F5 bind reproducibly and specifically to the DEFA5 epitope with moderate nanomolar affinity. Despite targeting the same epitope region, 1A8 demonstrated significantly higher binding affinity than 4F5. Statistical analysis using an unpaired two-tailed *t*-test showed a significant difference in binding strength between the two antibodies (*p* = 0.0409, *p* < 0.05), suggesting distinct paratope-epitope interaction dynamics.

### 3.7. Sequence analysis of 1A8 and 4F5 monoclonal antibodies

To determine the molecular basis of antigen recognition by the 1A8 and 4F5 monoclonal antibodies, the variable regions of both the heavy (VH) and light (VL) chains were amplified and sequenced. PCR amplification of the VH and VL regions was performed using primer sets specific for conserved framework regions. To ensure complete and accurate sequence coverage, 3′ rapid amplification of cDNA ends (3′ RACE-PCR) was also performed. The amplified products were purified and subjected to Sanger sequencing. Sequence alignment and analysis of both conventional PCR and 3’RACE-PCR products revealed that both 1A8 and 4F5 antibodies shared identical VH and VL nucleotide and deduced amino acid sequences (Tables 2 and 3), indicating that these two monoclonal antibodies likely originate from the same B cell clone or possess convergent recombination events resulting in the same antigen-binding site. Specifically, the CDRs in the VH chain were identified as CDR1 with the sequence GYTFFDYE, CDR2 with the sequence IHPGSGDT, and CDR3 with the sequence TREGTVVAPFDY. In the VL chain, CDR1 was defined as QSIVHSNGNTY, CDR2 as KVS, and CDR3 as FQGSYVPYT. These CDR sequences are highlighted in bold red text within the tables to indicate their critical role in antigen binding. The differential antigen recognition patterns observed between 1A8 and 4F5, such as their differential reactivity in the presence of a sc-53,997 antibody suggest that these differences are not due to variations in their variable regions, but may instead result from differences in antibody post-translational modifications. Affinity-purified recombinant 1A8 and 4F5 antibodies, expressed in HEK293 cells, showed binding affinities to recombinant DEFA5 (indirect ELISA) and DEFA5 in IBD biopsy samples (sandwich ELISA) comparable to the parental mouse monoclonal antibodies, confirming the sequencing data (data not shown)

### 3.8. Structural and binding analysis of DEFA5 epitope and 1A8/4F5 antibodies

**Fig. 6.**
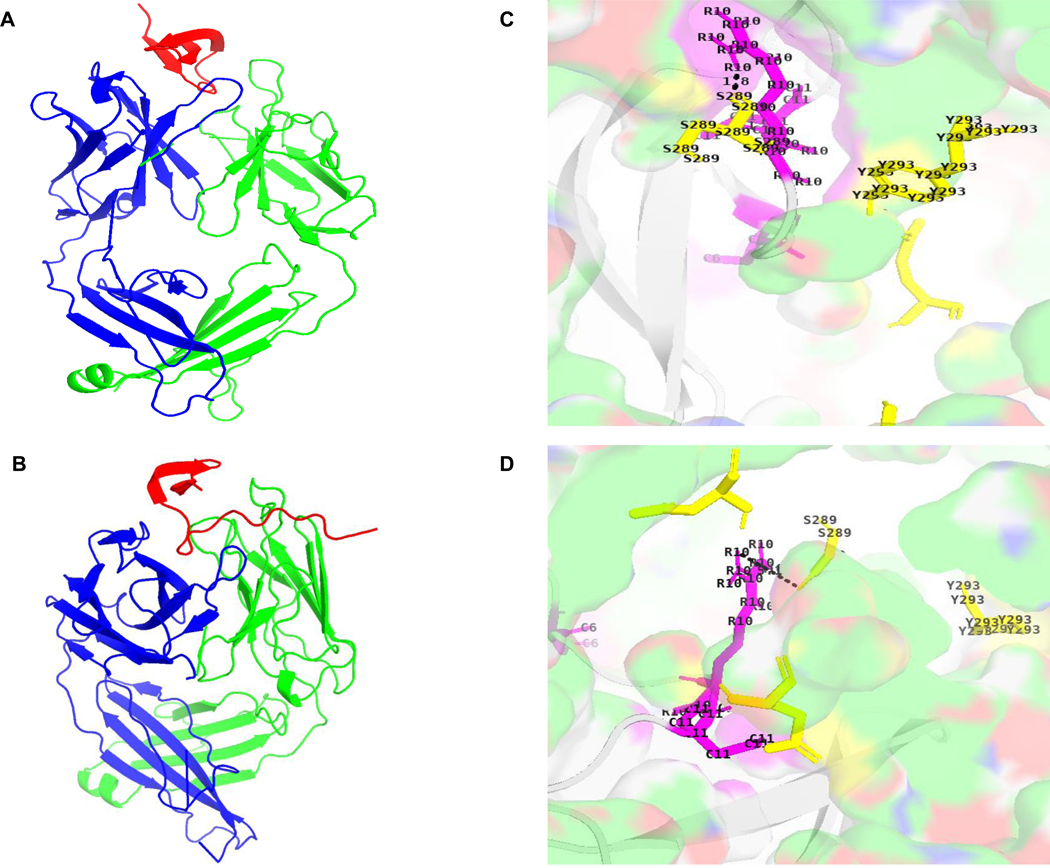
Structural models of 1A8 and 4F5 Fab-DEFA5 complexes generated by HADDOCK docking. (A) 1A8 Fab complex, with DEFA5 (red) bound primarily by the heavy chain (VH, blue). (B) 4F5 Fab complex, with DEFA5 (red) bound mainly by the light chain (VL, green). (C) Key 1A8–epitope interface, highlighting interactions with residues R10, C6, and C11 (yellow sticks) and VH residues S289 and Y293 (green). (D) Key 4F5–epitope interface, showing interactions with the same epitope residues (yellow sticks) and VL residues S289 and Y293 (green). Residue R10 plays a central role in both complexes.

To structurally contextualize the epitope RATCYCRTGRCAT, we mapped its sequence onto the available experimental NMR structure of DEFA5 (PDB ID: 1ZMP), which encompasses residues 63–94, with R62 excluded due to propeptide cleavage. This analysis revealed that the epitope is distributed across several structural elements of the protein’s core (Fig. 5C). Specifically, residues A2 and T3 are located on strand β2, while C4, Y5, and C6 are situated on the β2-β3 loop, which contains a disulfide-bonded cysteine. Furthermore, residues R7, T8, G9, and R10 are located on strand β3, and finally, residues C11, A12, and T13 are part of the C-terminal region. The N-terminal arginine (R1, corresponding to R62 in the full protein) was absent from this experimental model. This confirms that the epitope is conformational, formed by the three-dimensional folding of the peptide backbone that brings these disparate sequence regions into proximity. Mapping of the complete epitope onto an AlphaFold2-predicted full-length model (AF-Q01523-F1) confirmed the presence of all residues and their structural locations, predicting that the missing R1 residue resides in a disordered N-terminal region preceding strand β2 (Fig. 5D). Red and yellow regions denote the mature chain portion (residues 63–74), while magenta marks R62 from the propeptide-mature junction, collectively representing the full RATCYCRTGRCAT sequence. Docking via HADDOCK 2.4 incorporated active restraints from mutagenesis data, defining R10, C6, and C11 on DEFA5’s epitope RATCYCRTGRCAT as key sites, alongside CDR regions on both antibodies (VH: GYTFFDYE, IHPGSGDT, TREGTVVAPFDY; VL: QSIVHSNGNTY, KVS, FQGSYVPYT), revealing compact binding modes where DEFA5 adopts a looped conformation embraced by the Fab (Fig. 6A and B). PDBePISA analysis of high-ranking clusters showed interfaces dominated by salt bridges (R10 with S289/Y293), hydrophobic contacts involving C6 and C11, and buried surface areas of ~1718 Å^2^ for 1A8 and ~ 1909 Å^2^ for 4F5, with detailed residue interactions depicted in Fig. 6C and Fig. 6D. Prodigy-predicted Kd of 8.6 × 10^−10^ M (0.86 nM) for 1A8 and 1.03 × 10^−9^ M (1.03 nM) for 4F5 reflect strong nanomolar affinities, driven by 11 hydrogen bonds in 1A8 and 10 in 4F5 aligned with 1A8’s higher affinity despite a smaller buried surface area (~1718 Å^2^ vs. ~1909 Å^2^ for 4F5) highlights the role of specific interactions and binding poses. These computational estimates are tighter than the experimental ELISA Kd values of 43.83 nM for 1A8 and 54.97 nM for 4F5, yielding fold differences of approximately 51-fold and 53-fold, respectively, a common discrepancy in docking-based predictions that often overestimate affinity due to approximations in solvation, conformational entropy, and dynamic effects. Notably, despite identical hybridoma sequencing from 5’ RACE and 3’ RACE PCR, the models indicated subtle differences in binding poses, with DEFA5 primarily engaging the VH domain (blue region) in 1A8 (Fig. 6A) versus the VL domain (green region) in 4F5 (Fig. 6B), suggesting phenotypic variations in domain contributions.

### 3.9. Intact ESI-MS reveals divergent Fc glycosylation profiles between 1A8 and 4F5antibodies

**Fig. 7.**
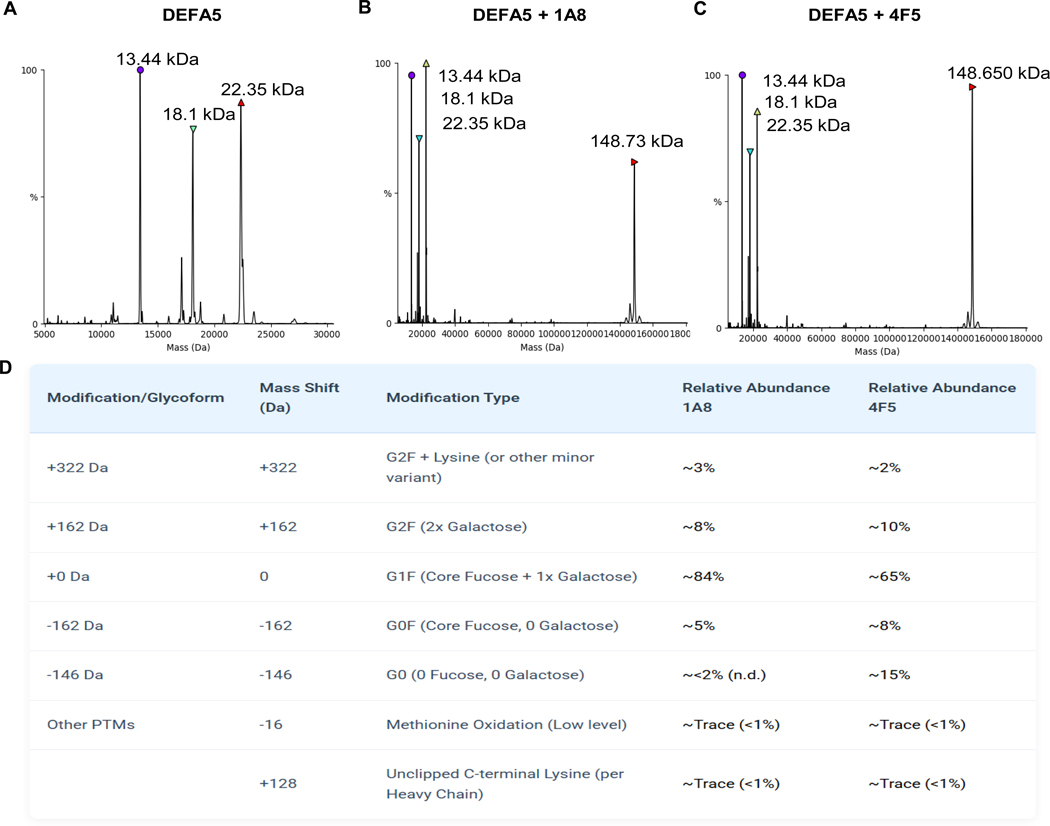
Intact ESI-MS and glycoform analysis of monoclonal antibodies 1A8 and 4F5. (A) Deconvoluted spectrum of DEFA5 alone showing peaks at 13.44 kDa, 18.1 kDa, and 22.35 kDa (B) Deconvoluted intact mass spectrum acquired for 1A8 in the presence of DEFA5, revealing a major antibody peak at ~148.73 kDa. (C) Deconvoluted intact mass spectrum acquired for 4F5 in the presence of DEFA5, displaying a dominant antibody peak at ~148.65 kDa. (D) Summary of glycoform and post-translational modification (PTM) assignments for 1A8 and 4F5 antibodies based on intact ESI-MS deconvolution.

Electrospray ionization mass spectrometry (ESI-MS) was performed to assess potential complex formation between *E. coli* expressed DEFA5 and monoclonal antibodies 1A8 and 4F5. Spectra were acquired for DEFA5 alone (Fig. 7A), DEFA5 in complex with 1A8 (Fig. 7B), and DEFA5 in complex with 4F5 (Fig. 7C). No new peaks corresponding to DEFA5-antibody complexes were observed in either antibody mixture, indicating an absence of stable complex formation in solution. Notably, intact masses of 1A8 and 4F5 antibodies were measured as ~148.73 kDa and ~ 148.65 kDa, respectively, consistent with a molecular weight difference of ~80 Da, potentially reflecting a post-translational modification or minor sequence variation (Fig. 7B and C). Deconvolution of the intact mass spectra revealed a striking difference in glycosylation heterogeneity between the two clones (Fig. 7D). The 1A8 antibody exhibited a homogeneous profile, with its mass spectrum dominated by a primary peak corresponding to the G1F glycoform. In contrast, the 4F5 antibody displayed a more heterogeneous profile, characterized by a significantly reduced abundance of the G1F glycoform and a substantial emergence of afucosylated (G0) species. This ~15 % abundance of G0 glycoforms in 4F5 compared to <2 % in 1A8 represents a greater than 7-fold increase and is the defining post-translational difference between the two clones. The abundances of other glycoforms (G0F, G2F) and low-level post-translational modifications (oxidation, unclipped lysine) were comparable between the two clones. ESI-MS analysis of recombinant DEFA5 revealed three major species with molecular weights of approximately 13.44 kDa, 18.1 kDa, and 22.35 kDa. These findings support the presence of multiple DEFA5 isoforms, which may have distinct biological activities or relevance in downstream applications. The 22.35 kDa species aligns with the calculated molecular weight of recombinant construct (22.35 kDa), while the 13.44 kDa and 18.1 kDa species suggest processed intermediates, possibly arising from partial proteolytic cleavage in the recombinant system.

## 4. Discussion

Accurate subclassification of IBD, notably UC, CC, and the inexact diagnosis of IC remains a significant diagnostic challenge because of presenting overlapping clinical and histological features [19,20]. The antimicrobial peptide DEFA5 has emerged as a promising biomarker for distinguishing IBD subtypes because its expression is restricted to colonic IBD conditions and associated with Paneth ileal-cell metaplasia, a feature more common in Crohn’s colitis than ulcerative colitis [4,5]. However, currently available DEFA5 commercial antibodies, often lack well-defined epitope specificity or exhibit inconsistent performance across tissue types and assay platforms [4,6–8]. These limitations underscore the need for novel, rigorously characterized monoclonal antibodies with defined binding properties suitable for diagnostic applications. In the present study, we comprehensively characterized two novel mouse mAbs, 1A8 and 4F5, that specifically recognize human DEFA5. By integrating hydrogen/deuterium exchange mass spectrometry (HDX-MS), peptide mapping, surface plasmon resonance (SPR), and paratope analysis, we sought to elucidate the molecular basis of antibody-DEFA5 recognition and evaluate the diagnostic potential of these clones.

HDX-MS analysis demonstrated that both 1A8 and 4F5 binding led to diffuse protection with partial protection patterns in DEFA5, consistent with conformational stabilization or indirect shielding effects rather than direct protection of a discrete epitope. These findings align with prior studies showing that antibodies binding to small, disulfide-rich peptides like defensins often induce allosteric stabilization rather than engaging a single linear or conformational epitope [21,22]. The structural compactness of DEFA5, with its characteristic disulfide array, likely limits the accessibility of individual residues for HDX protection. Indeed, HDX-MS has been shown to detect protection at sites influenced by allosteric effects or global stabilization, complicating precise epitope localization in such contexts [23–25].

To overcome these limitations, we employed peptide-based mapping. Peptide mapping is particularly useful for small and structurally constrained antigens like DEFA5, where conformational or topological factors may limit the resolution of structural epitope mapping techniques. This approach relies on the binding of antibodies to overlapping synthetic peptides spanning the antigen’s primary sequence, enabling detection of continuous (linear) or segmental binding regions with high sensitivity [26,27]. Peptide-based mapping successfully delineated localized linear binding regions that were not fully resolved by HDX-MS. This highlights the complementary nature of linear peptide mapping for detecting continuous epitopes, particularly in the context of structurally rigid antigens like DEFA5. Importantly, our peptide-based mapping identified RATCYCRTGRCAT as a key linear sequence contributing to antibody recognition. In addition to epitope mapping, we used surface plasmon resonance (SPR) to characterize the binding kinetics and stoichiometry of 1A8 and 4F5 with peptide epitope. SPR is a gold-standard, label-free method for quantifying real-time biomolecular interactions, allowing precise determination of association (ka), dissociation (kd), and equilibrium constants (KD), as well as providing insights into binding stoichiometry and avidity [28,29]. Surface plasmon resonance (SPR) analysis provided quantitative insight into the binding kinetics and stoichiometry of both mAbs, revealing that despite targeting overlapping regions, 1A8 and 4F5 exhibited distinct affinities and kinetics. This peptide presents a single binding site, and the observed 1:1 stoichiometry is consistent with monovalent engagement of this epitope by the antibody’s Fab region. These findings support the conclusion that 1A8 and 4F5 engage DEFA5 through specific, non-redundant binding sites without evidence of cross-linking or multivalent binding, which aligns with their potential utility in diagnostic assays requiring precise antigen recognition. Although SPR successfully validated the RATCYCRTGRCAT motif as the shared binding site for both 1A8 and 4F5 antibodies using synthetic peptide, attempts to measure binding kinetics using full-length DEFA5 directly immobilized on the SPR sensor chip did not yield interpretable responses (data not shown). This likely reflects challenges related to the conformation, orientation, or steric hindrance of DEFA5 when immobilized to the chip surface, factors known to impair effective epitope presentation and hinder reliable binding measurements in SPR assays [30–32]. Due to these technical limitations, indirect ELISA was employed as an alternative approach to assess binding affinity under solution phase conditions. Indirect ELISA using full-length DEFA5 yielded KD values in the moderate nanomolar range (42.15–45.52 nM) for 1A8 and (53.37–56.57 nM) for 4F5. The observed difference between SPR and ELISA-derived values is consistent with previous findings, where SPR is known to measure monovalent real-time interactions in a controlled flow environment, while ELISA reflects equilibrium binding to immobilized full-length antigens and may be influenced by multivalent effects or steric hindrance [29–31]. Despite these methodological distinctions, both assays consistently demonstrate that 1A8 exhibits higher affinity than 4F5 for the same epitope, likely due to subtle paratope differences. This reinforces the functional importance of the RATCYCRTGRCAT region as a shared epitope for both antibodies and underscores the value of orthogonal validation strategies in antibody characterization [22,33]. The structural analyses support our findings on epitope recognition by 1A8 and 4F5 antibodies, crucial for diagnostics in IBD. The disulfide-bonded cysteines within the mature chain (residues 63–74) of the RATCYCRTGRCAT epitope provide conformational stability, ensuring a consistent structure for antibody binding, which is vital for reliable diagnostic sensitivity. The hybridoma sequencing revealed that both antibodies utilize multiple complementarity-determining regions (CDRs) from both the heavy and light chains to engage DEFA5, consistent with a structurally distributed binding interface. Such multivalent interactions likely contribute to the differential binding properties observed and may enhance diagnostic performance by providing greater resilience against minor epitope alterations. Notably, the ~51–53-fold discrepancy between Prodigy-predicted Kd values (0.86 nM for 1A8, 1.03 nM for 4F5) and ELISA-measured Kd values (43.83 nM for 1A8, 54.97 nM for 4F5) suggests that computational models may underestimate entropic or solvent effects influencing binding affinity, potentially amplifying the observed differences in binding kinetics between the antibodies [34]. This observation is consistent with previous reports indicating that subtle structural or conformational differences often outside the CDRs or within their three-dimensional arrangement can significantly influence antibody-antigen binding kinetics and affinity, even among clonally related antibodies [35–37]. High-resolution mass spectrometry analysis of the purified antibodies revealed an 80 Da molecular weight difference between 1A8 and 4F5. This mass discrepancy likely corresponds to a minor sequence variation or a post-translational modification (PTM), such as oxidation, methylation, or deamidation, located within or proximal to one of the CDR loops. This mass discrepancy, combined with the observed glycoform and PTM profiles may subtly alter the structural conformation, charge distribution, or hydrophobicity of the antigen-binding site, resulting in differences in antigen recognition and affinity [38–43]. The glycoform analysis showed 1A8 with a dominant G1F glycoform (~84 %) and lower G0 (~2 %), while 4F5 exhibited a higher G0 (~15 %) and lower G1F (~65 %), indicating greater afucosylation in 4F5. This afucosylation, known to enhance antibody-dependent cellular cytotoxicity (ADCC) [44], contrasts with 1A8’s more uniform glycosylation, which may contribute to its superior binding strength and kinetic stability [45]. The presence of trace PTMs like methionine oxidation (−16 Da) and unclipped C-terminal lysine (+128 Da) at <1 % in both antibodies suggests minimal degradation impact. The 80 Da difference, potentially a PTM such as deamidation (+1 Da per site, though cumulative effects or other modifications could approximate 80 Da), along-side glycoform variations, likely underlies the observed functional differences, despite nearly identical paratope sequences. These findings collectively demonstrate that although 1A8 and 4F5 recognize a shared epitope on DEFA5, they do so with distinct binding properties that can influence assay performance. This distinction has important implications for **serological and tissue based diagnostics**, as each antibody may perform optimally under different conditions or sample types when used to distinguish IBD subtypes, including Ulcerative colitis, Crohn’s colitis, and Indeterminate colitis.

## 5. Conclusions

Our data provide a comprehensive characterization of DEFA5-antibody interactions. The complementary use of HDX-MS, peptide mapping, SPR, and paratope sequencing has enabled a deeper understanding of the molecular basis for 1A8 and 4F5 binding. HADDOCK models further reveal subtle binding pose differences, with DEFA5 engaging the VH domain in 1A8 and the VL domain in 4F5, supported by 11 versus 10 hydrogen bonds and buried surface areas of 1718 Å^2^ and 1909 Å^2^, respectively. These findings support the development of more precise DEFA5-targeted diagnostic assays, which could improve IBD subtype classification and assist in resolving cases of IC into authentic UC and CC or a novel colitis with different pathological characteristics.

## Supplementary Material

NIHMS2068347_Supplementary Figs S1 - S5

## Figures and Tables

**Table 1:** Comprehensive peptide library spanning the full-length DEFA5 protein. A set of 64 synthetic peptides, each 15 amino acids in length, was designed to systematically cover the entire DEFA5 sequence. Each peptide overlaps the previous by 12 residues, with a 3-residue offset, providing full epitope coverage with high resolution for antibody-binding analysis. This library was used for ELISA-based epitope mapping and downstream functional validation studies.

No.	Peptide sequence	No.	Peptide sequence	No.	Peptide sequence

1	GSSHHHHHHGSGLP	22	PLRRLMEAFAKRQGK	43	NLYFQERADEATTQK
2	HHHHHHGSGLVPRGS	23	RLMEAFAKRQGKEMD	44	FQERADEATTQKQSG
3	HHHGSGLVPRGSASMS	24	EAFAKRQGKEMDSLR	45	RADEATTQKQSGEDN
4	GSGLVPRGSASMSDS	25	AKRQGKEMDSLRFLY	46	EATTQKQSGEDNQDL
5	LVPRGSASMSDSSEVN	26	QGKEMDSLRFLYDGI	47	TQKQSGEDNQDLAIS
6	RGSASMSDSSEVNQEA	27	EMDSLRFLYDGRIQ	48	QSGEDNQDLAISFAG
7	ASMSDSSEVNQEAKPE	28	SLRFLYDGIRIQADQ	49	EDNQDLAISFAGNGL
8	SDSEVNQEAKPEVKP	29	FLYDGIRIQADQTPE	50	QDLAISFAGNGLSAL
9	EVNQEAKPEVKPEVK	30	DGIRIQADQTPEDLD	51	AISFAGNGLSALRTS
10	QEAKPEVKPEVKPET	31	RIQADQTPEDLDMED	52	FAGNGLSALRTSGSQ
11	KPEVKPEVKPETHIN	32	ADQTPEDLDMEDNDI	53	NGLSALRTSGSQQARA
12	VKPEVKPETHINLKV	33	TPEDLDMEDNDIIEA	54	SALRTSGSQQARATCY
13	EVKPETHINLKVSDS	34	DLDMEDNDIIEAHRE	55	RTSGSQQARATCYCRT
14	PETHINLKVSDSGSSE	35	MEDNDIIEAHREQIG	56	GSQQARATCYCRTGR
15	HINLKVSDSGSSEIFF	36	NDIIEAHREQIGGRQ	57	ARATCYCRTGRCATR
16	LKVSDSGSSEIFFKIK	37	IEAHREQIGGRQAGS	58	TCYCRTGRCATRESL
17	SDSGSSEIFFKIKKTT	38	HREQIGGRQAGSGSE	59	CRTGRCATRESLSGV
18	SSEIFFKIKKTTPLR	39	QIGGRQAGSGSENLY	60	GRCATRESLSGVCEI
19	IFFKIKKTTPLRRLM	40	GRQAGSGSENLYFQE	61	ATRESLSGVCEISGR
20	KIKKTTPLRRLMEAF	41	AGSGSENLYFQERADE	62	ESLSGVCEISGRLYR
21	KTTPLRRLMEAFAKR	42	GSENLYFQERADEATT	63	SGVCEISGRLYRLCC
				64	CEISGRLYRLCCR

**Table 2.** 5′ RACE PCR nucleotide sequences and IMGT-converted amino acid sequences of the VH and VL chains of monoclonal antibodies 1A8 and 4F5. Both antibodies showed identical sequences. The Complementarity-determining regions (CDR1, CDR2, and CDR3) are highlighted in red.

Chain	5’RACE PCR Nucleotide Sequence	IMGT Converted Amino Acid Sequence

**VH**	ATGGAATGGAGCTGGGTCTTTCTCTTCCTCCTGTCAGTAACTGCAGGTGTCCAATCCCAGGTTCAACTGCAGCAGTCTGGGGCTGAGCTGGTGAGGCCTGGGGCTTCAGTGAAGATGTCCTGCAAGGCTTTGGGCTACACATTTTTTGACTATGAAATGCACTGGATGAAGCAGACACCTGTGTATGGCCTGGAATGGATTGGAGCTATTCATCCAGGAAGTGGTGATACTGCCTACAATCAGAAGTTCAAGGGCAAGGCCACACTGACTGCAGACAAATCCTCCAACACAGCCTACATGGAGCTCAGCAGCCTGACATCTGAGGACTCTGCTGTCTATTACTGTACAAGAGAGGGTACGGTAGTAGCACCCTTTGACTACTGGGGCCAAGGCACCACTCTCACAGTCTCCTCAGCCAAAACGACACCCCCATCTGTCTATCCACTGGCCCCTGGATCTGCTGCCCAAACTAACTCCATGGTGACCCTGGGATGCCTGGTCAAGGGCTATTTCCCTGAGCCAGTGACAGTGACCTGGAACTCTGGATCCCTGTCCAGCGGTGTGCACACCTTCCCAGCTGTCCTGCAGTCTGACCTCTACACTCTGAGCAGCTCAGTGACTGTCCCCTCCAGC	MEWSVWLFFLSVTAGVQSQLQQSGAELVRPGASVKMSCKALGYTFFDYEMHWMKQTPVYGLEWIGAIHPGSGDTAYNQKFKGKATLTADKSSNTAYMELSSLTSEDSAVYYCTREGTVVAPFDYWGQGTTLTVSSAKTTPPSVYPLAPGSAAQTNSMVTLGCLVKGYFPEPVTVTWNSGSLSSGVHTFPAVLQSDLYTLSSSVTVPSS
**VL**	ATGAAGTTGCCTGTTAGGCTGTTGGTGCTGATGTTCTGGATTCCTGCTTCCAGCAGTGATGTTTTGATGACCCAAACTCCACTCTCCCTGCCTGTCAGCCTTGGAGATCAAGCCTCCATCTCTTGCAGATCTAGTCAGAGTATTGTACATAGTAATGGAAACACCTATTTAGAATGGTACCTTCAGAAACCAGGCCAGTCTCCAAAACTCCTGATAAACAAAGTTTCCAACCGATTTTCTGGGGTCCCAGACAGGTTCAGTGGCAGTGGATCAGGGACAGATTTCACACTCAAGATCAGCAGAGTGGAGGCTGAGGATCTGGGAGTTTATTACTGCTTTCAAGGTTCATATGTTCCGTACACGTTCGGAGGGGGGACCAAGCTGGAAATAAAACGGGCTGATGCTGCACCAACTGTATCCATCTTCCCACCATCCAGTGAGCAGTTAACATCTGGAGGTGCCTCAGTCGTGTGCTTCTTGAACAACTTCTACCCCAAAGACATCAATGTCAAGTGGAAGATTGATGGCAGTGAACGACAAAATGGCGTCCTGAACAGTTGGACTGATCAGGACAGCAAAGACAGCACCTACAGCATGAGCAGCACCCTCACGTTGACCAAG	MKLPVRLLVLMFWIPASSSDVLMTQTPLSLPVSLGDQASISCRSSQSIVHSNGNTYLEWYLQKPGQSPKLLINKVSNRFSGVPDRFSGSGSGTDFTLKISRVEAEDLGVYYCFQGSYVPYTFGGGTKLEIKRADAAPTVSIFPPSSEQLTSGGASVVCFLNNFYPKDINVKWKIDGSERQNGVLNSWTDQDSKDSTYSMSSTLTLTK

**Table 3.** 3′ RACE PCR–derived IMGT-converted amino acid sequences of monoclonal antibodies 1A8 and 4F5. Both antibodies exhibited identical sequences. The CDR1, CDR2, and CDR3 regions are highlighted in red.

Chain	3’RACE PCR Derived IMGT Converted Amino Acid Sequence

VH	MDPKGSLSWRILLFLSLAFELSYGQVQLQQSGAELVRPGASVKMSCKALGYTFFDYEMHWMKQTPVYGLEWIGAIHPGSGDTAYNQKFKGKATLTADKSSNTAYMELSSLTSEDSAVYYCTREGTVVAPFDYWGQGTTLTVSSAKTTPPSVYPLAPGSAAQTNSMVTLGCLVKGYFPEPVTVTWNSGSLSSGVHTFPAVLQSDLYTLSSSVTVPSSTWPSETVTCNVAHPASSSTKVDKKIVPRDCGCKPCICTVPEVSSVFIFPPKPKDVLTITLTPKVTCVVVDISKDDPEVQFSWFVDDVEVHTAQTQPREEQFNSTFRSVSELPIMHQDWLNGKEFKCRVNSAAFPAPIEKTISKTKGRPKAPQVYTIPPPKEQMAKDKVSLTCMITDFFPEDITEVWQWNGQPAENYKNT PIMDTDGSYFVYSKLNVQKSNWEAGNTFTCSVLHEGLHNHHTEKSLSHPG
VL	METDTLLLWVLLLWVPGSTGDVLMTQTPLSPVSLGDQASISCRSSQSIVHSNGNTYLEWYLQKPGQSPKLLINKVSNRFSGVPDRFSGSGSGTDFTLKISRVEAEDLGVYYCFQGSYVPYTFGGGTKLEIKRADAAPTVSIFPPSSEQLTSGGASWCFLNNFYPKDINVKWKIDGSERQNGVLNSWTDQDSKDSTYSMSSTLTLTKDEYERHSYTCEATHKTSTSPIVKSFNRNEC

## Data Availability

Data is available upon request. The data that support the findings of this study are available from the corresponding authors.

## Appendix A. Supplementary data

Supplementary data to this article can be found online at https://doi.org/10.1016/j.ijbiomac.2025.148024.
