# Supplementary material for: Structural and epitope characterization of anti-DEFA5 monoclonal antibodies clones 1A8 and 4F5 for inflammatory bowel disease subtype diagnostics
Source: Int J Biol Macromol. Author manuscript; Available in PMC 2026 Jan 16. (PMC12809912; doi:10.1016/j.ijbiomac.2025.148024)
Supplement: NIHMS2068347_Supplementary Figs S1 - S5 [file NIHMS2068347-supplement-NIHMS2068347_Supplementary_Figs_S1_-_S5.pdf]

Structural and Epitope Characterization of Anti-DEFA5 Monoclonal Antibodies Clones 1A8 and 4F5 for Inflammatory Bowel Disease Subtype Diagnostics

supplementary figures

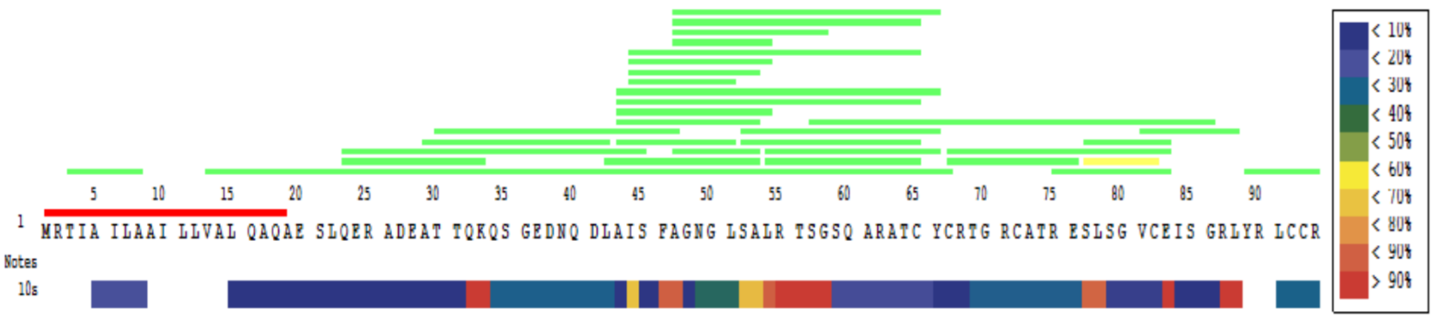

Fig S1. Peptide coverage map of DEFA5 in HDX-MS. The ProPharma DEFA5 sequence (UniProt Q01523, 94 amino acids) was used as the reference to achieve 93.6% coverage of the core DEFA5 sequence while avoiding complications from the recombinant construct's tag, linker, or fusion regions, which could produce poorly ionizing or irrelevant peptides. The N-terminal signal peptide (red line, canonical aa 1–19) was absent in the recombinant construct. Peptides appearing in the signal peptide region (red line) are mapping artifacts, peptide coverage starts from the residue of the propeptide DEFA5 (aa 23).

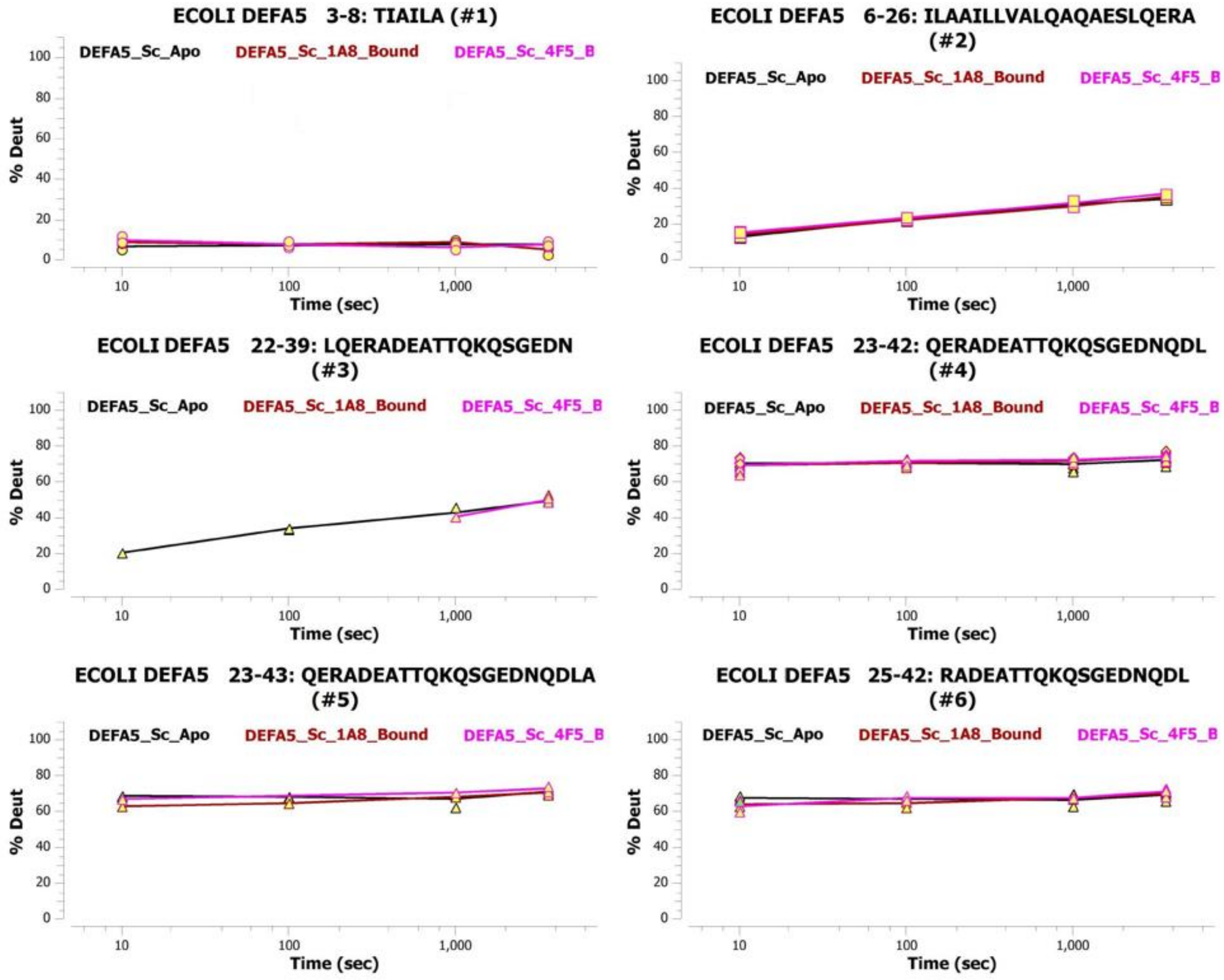

ECOLI DEFA5 25-43: RADEATTQKQSGEDNQDLA (#7)

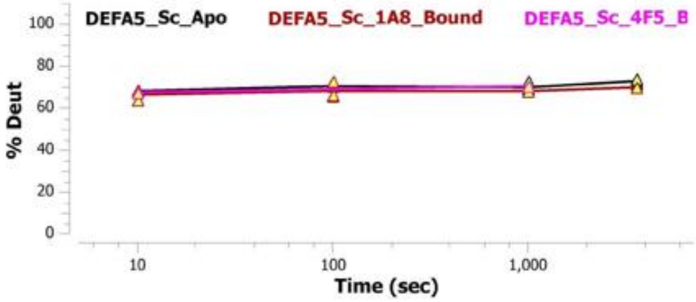

ECOLI DEFA5 29-42: ATTQKQSGEDNQDL (#8)

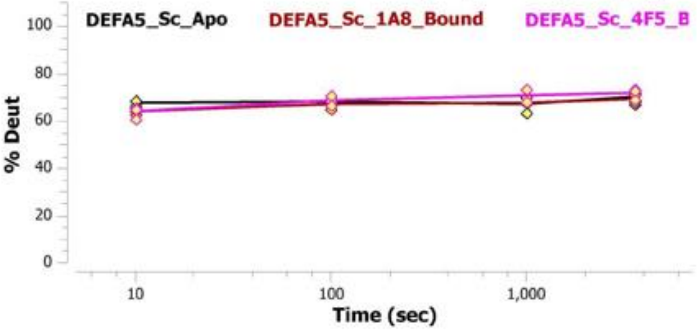

ECOLI DEFA5 29-43: ATTQKQSGEDNQDLA (#9)

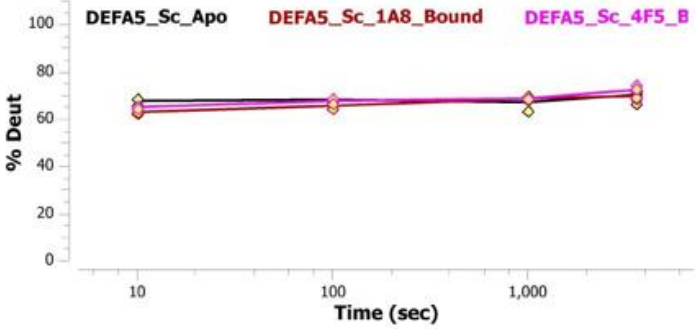

ECOLI DEFA5 37-60: EDNQDLAISFAGNGLSALRTSGSQ (#10)

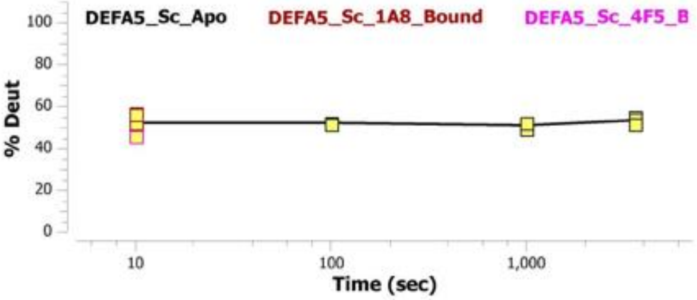

ECOLI DEFA5 41-94: GNGLSALRTSGSQARATCYCRTGRCATRESLSGVCEISGR (#11)

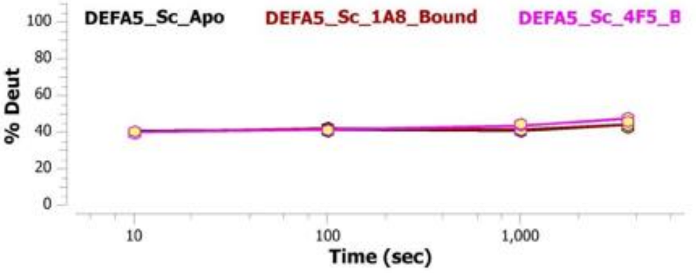

ECOLI DEFA5 42-53: LAISFAGNGLSA (#12)

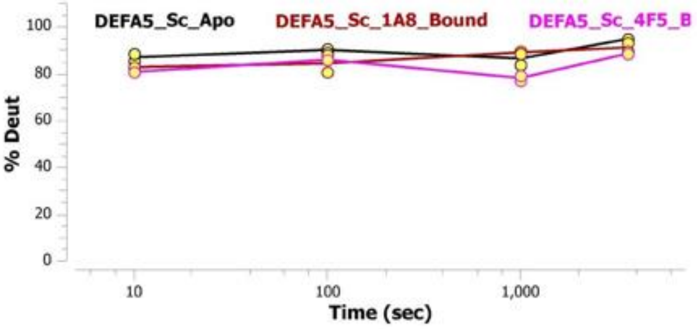

ECOLI DEFA5 43-51: AISFAGNGL (#13)

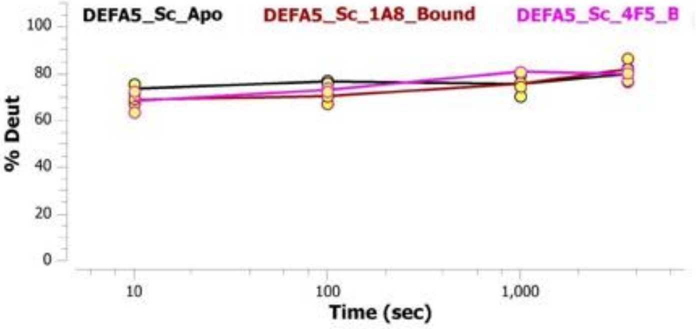

ECOLI DEFA5 43-53: AISFAGNGLSA (#14)

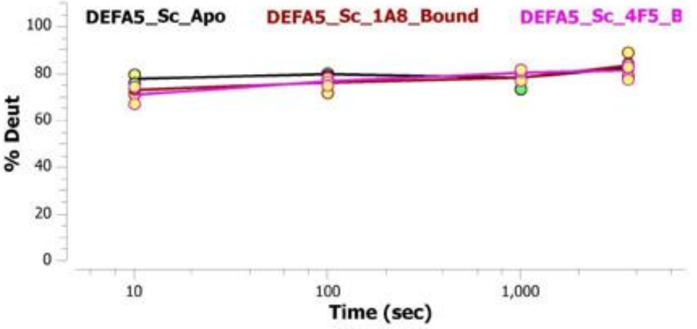

ECOLI DEFA5 43-54: AISFAGNGLSAL (#15)

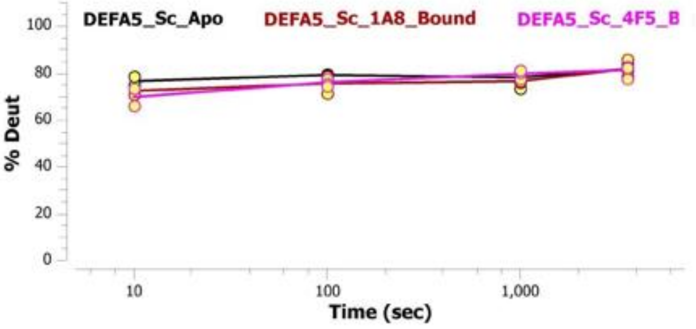

ECOLI DEFA5 43-65: AISFAGNGLSALRTSGSQARATC (#16)

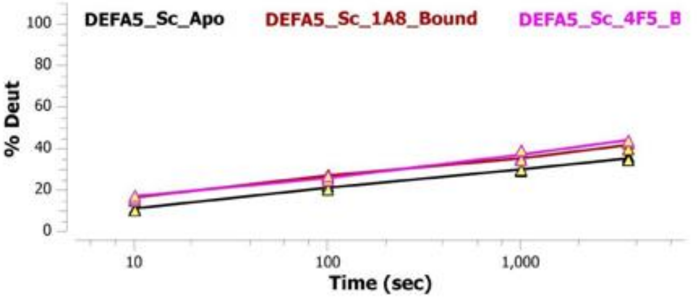

ECOLI DEFA5 44-51: ISFAGNGL (#17)

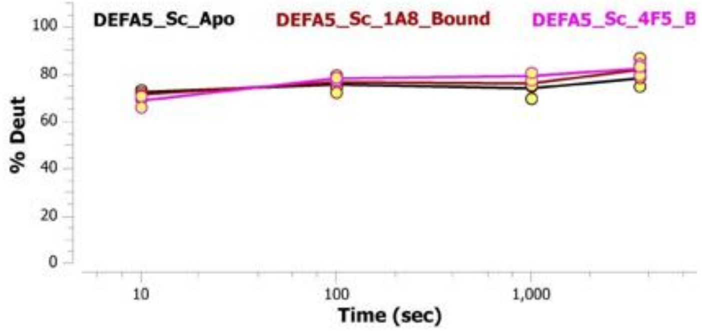

ECOLI DEFA5 44-53: ISFAGNGLSA (#18)

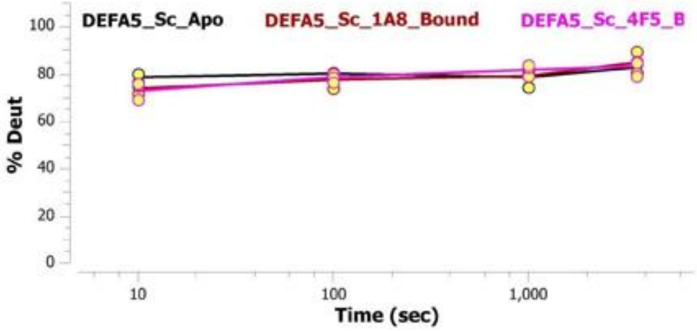

ECOLI DEFA5 44-54: ISFAGNGLSAL (#19)

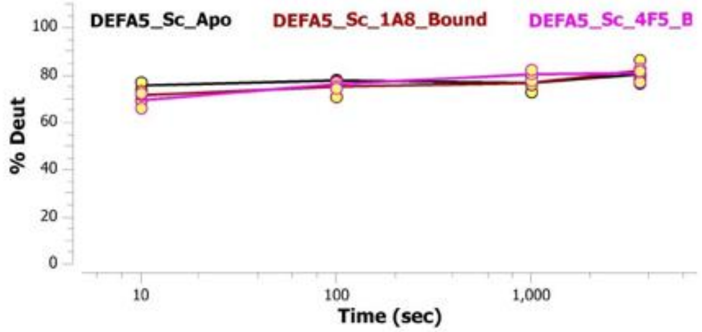

ECOLI DEFA5 45-54: SFAGNGLSAL (#20)

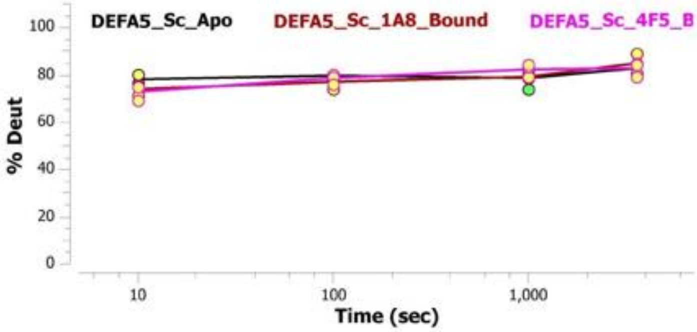

ECOLI DEFA5 47-53: AGNGLSA (#21)

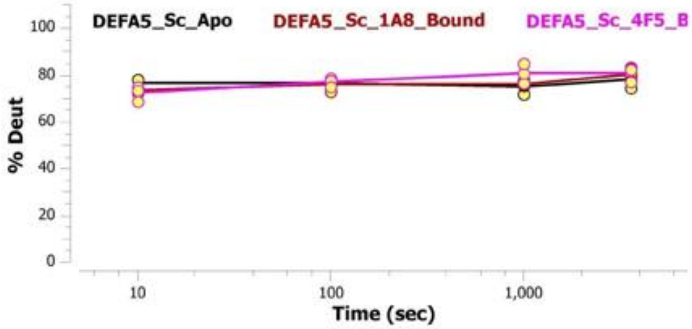

ECOLI DEFA5 47-54: AGNGLSAL (#22)

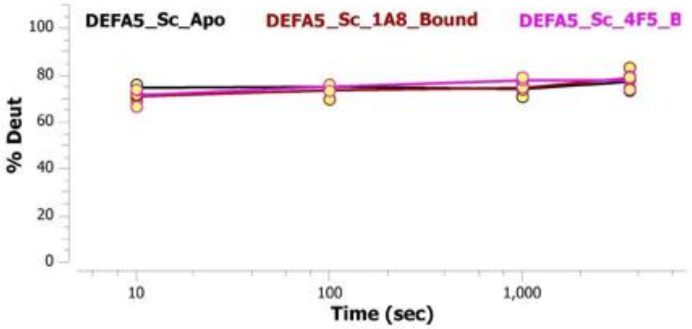

ECOLI DEFA5 47-65: AGNGLSALRTSGSQARATC (#23)

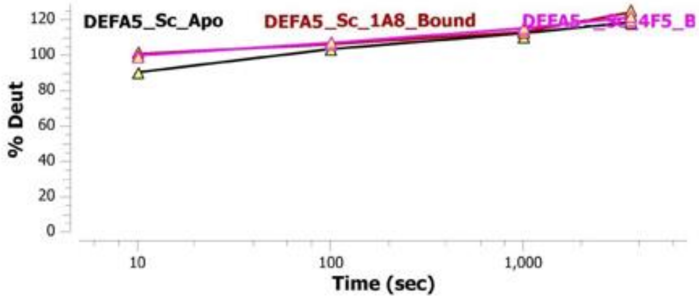

ECOLI DEFA5 52-66: SALRTSGSQARATCY (#24)

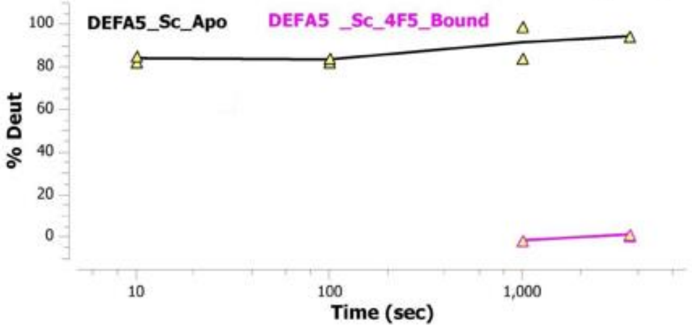

ECOLI DEFA5 54-65: LRTSGSQARATC (#25)

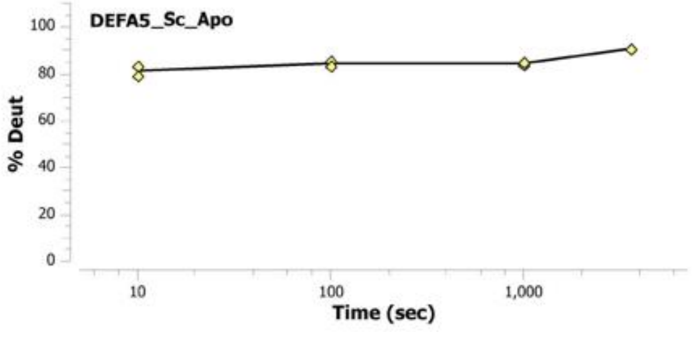

ECOLI DEFA5 54-66: LRTSGSQARATCY (#26)

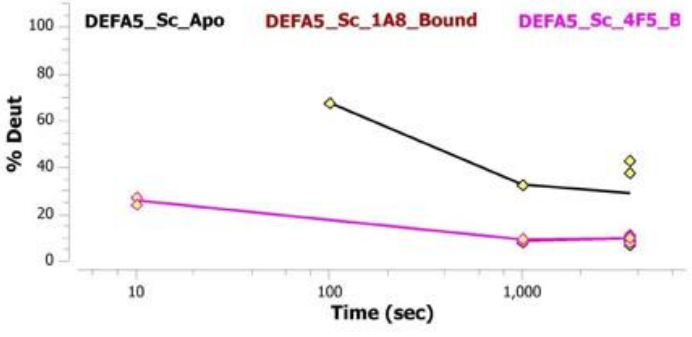

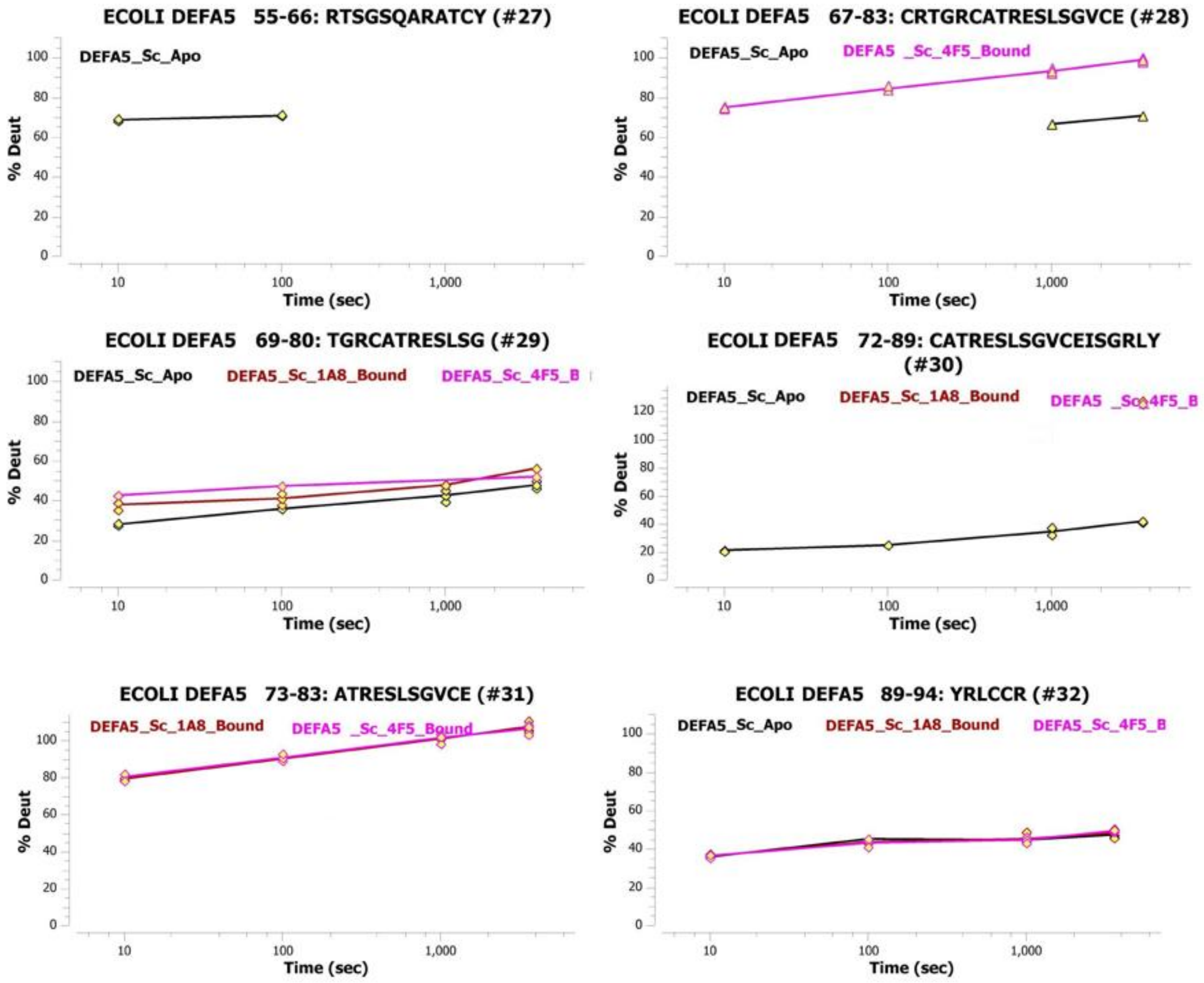

Fig. S2. Deuterium uptake plots compare DEFA5 in complex with the Santa Cruz DEFA5 antibody (sc-53997), and DEFA5 in complex with the Santa Cruz DEFA5 antibody plus either 1A8 or 4F5. The uptake plots show diffuse and partial protection upon 1A8 and 4F5 binding, localized to two overlapping peptides, LRTSGSQARATCY (residues 54-66) for 1A8 and SALRTSGSQARATCY (residues 52-66) for 4F5, with the latter extending two additional N-terminal residues.

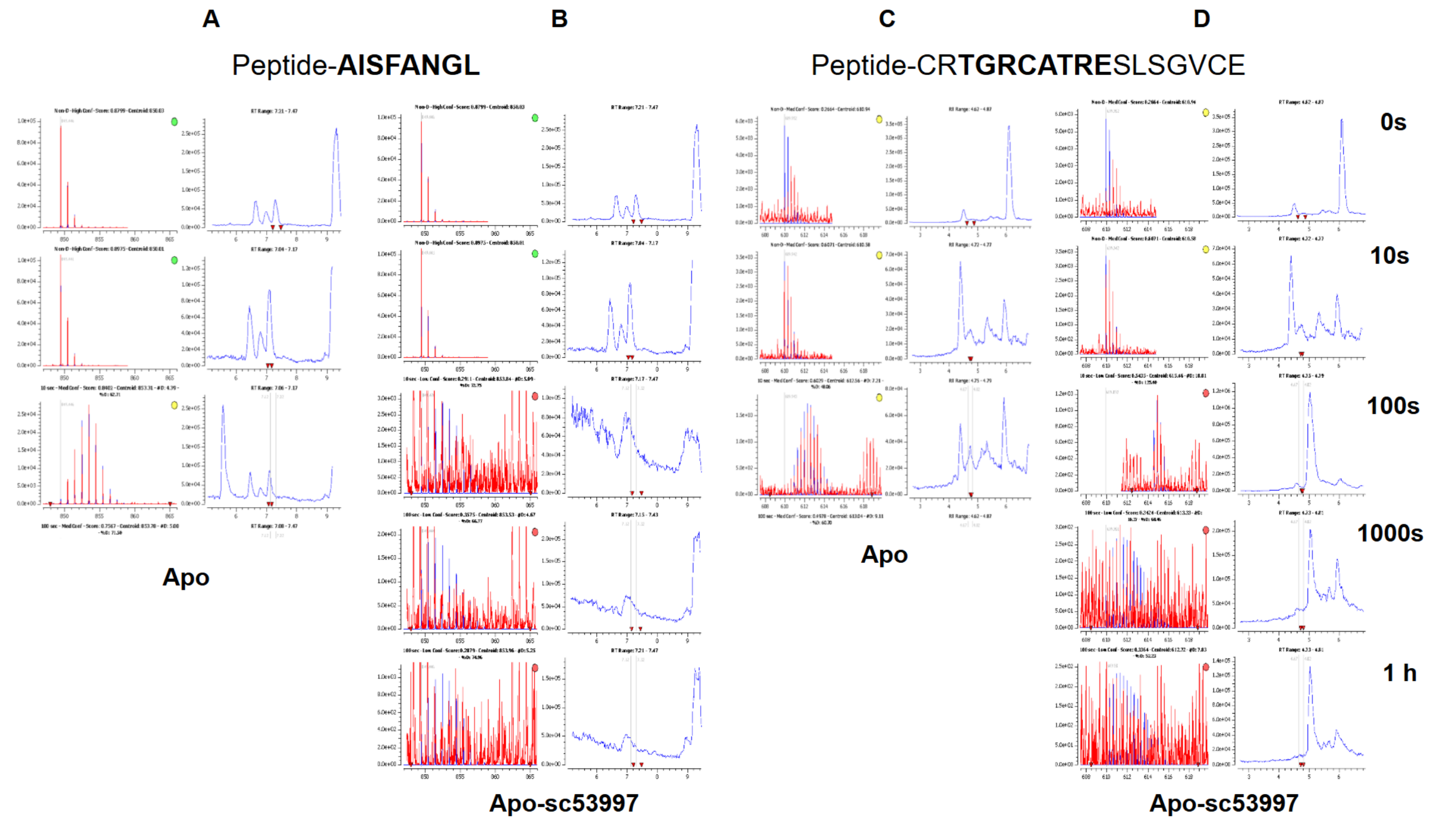

Fig S3. HDX-MS analysis of sc-53997 binding to DEFA5. Mass spectrometry spectra from HDX-MS experiments for DEFA5 peptides 67–83 (CR**TGRCATRE**SLSGVCE) and 43–51 (AISFANGL) at timepoints 0s, 10s, 100s, 1000s, and 1h, comparing apo (A, C) and Apo-sc53997 complex (B, D) states. Spectra for 67–83 show centroid shifts with reduced deuterium uptake (~48–61% D in complex vs. 68–125% D in apo) and EX1 to EX2 kinetic transition, confirming the sc-53997 epitope (core TGRCATRE). Spectra for 43–51 exhibit a similar EX1 to EX2 shift with uptake stabilizing at ~63–72% D, indicating allosteric stabilization. These data support sc-53997 antibody induced structural rearrangements enhancing 1A8/4F5 affinity by optimizing DEFA5 conformation. Due to stability issues, DEFA5_apo data for labeling times of 1000s and 1h deuterium uptake for these longer time points was predicted by exchange rate extrapolation using curve fitting of 10s and 100s apo spectra.

A

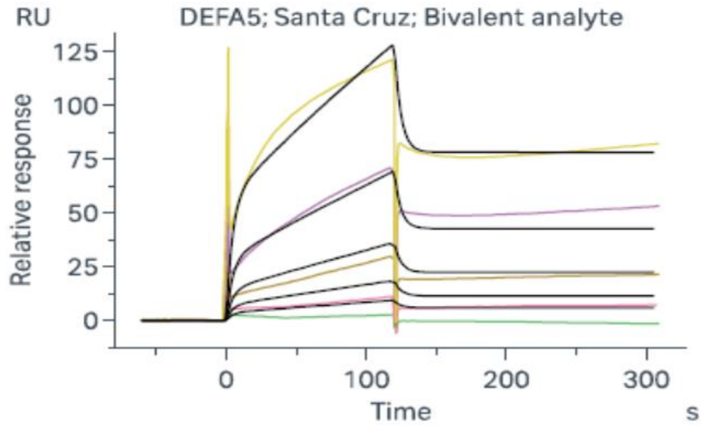

B

Ligand	Analyte	Chi^2 (RU^2)	ka1 (1/Ms)	kd1 (1/s)	ka2 (1/RUs)	kd2 (1/s)	KD (M)	Rmax (RU)
Santa Cruz	DEFA5	4.50E+01	7.71E+02	2.09E-01	8.65E-06	4.58E-06	2.71E-04	947.2

Fig S4. SPR analysis of sc-53997 binding to DEFA5. Sensogram of sc-53997 immobilized at 10 µg/mL (capture ~676.2 RU over 30s) with DEFA5 analyte at 372.02, 744.04, 1488.08, 2976.16, and 5952.32 nM over 120s association and 180s dissociation, analyzed with a bivalent analyte model. A. The sensogram shows high Rmax (947.2 RU) with slow dissociation despite a low molar ratio <0.1, suggesting a 1:2 stoichiometry (sc53997:DEFA5$_2$). The biphasic association curve and slow dissociation (~70 RU at highest concentration) suggest a bivalent binding model with possible oligomeric DEFA5 involvement. B. Summary of the Affinity data for sc-53997:DEFA5 interaction, highlighting bivalent binding and oligomerization, supporting structural rearrangements.

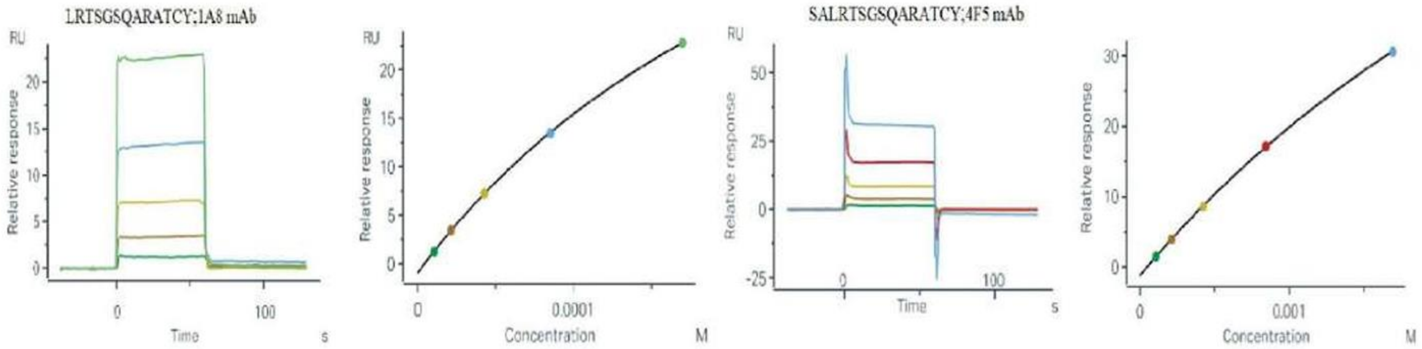

Ligand	Analyte	Chi^2 (RU^2)	ka (1/Ms)	kd (1/s)	KD (M)	Rmax (RU)
1A8mAb	LRTSGSQARATCY	5.14E-03	N/A	N/A	3.03E-04	66.1
4F5mAb	SALRTSGSQARATCY	1.49E-02	N/A	N/A	4.80E-03	122.1

Fig. S5. SPR sensorgrams showing weak binding interactions between antibody clones and DEFA5 peptides. Clone 1A8 exhibited weak binding to the peptide LRTSGSQARATCY (KD=303 µM), while clone 4F5 showed weak binding to the peptide SALRTSGSQARATCY (KD=4,800 µM), indicating that these segments alone are insufficient for specific, high affinity recognition and do not constitute complete functional epitopes, highlighting conformational flexibility in DEFA5.
